# Efficient Chirality-Induced Spin Selectivity in Self-Assembled Monolayers of Ru_2_^5^^+^ Paddlewheel Complexes

**DOI:** 10.1021/jacs.6c02811

**Published:** 2026-06-27

**Authors:** Isabel Coloma, Niccolò Giaconi, Francesco Parmeggiani, Thierry Buffeteau, Gilles Pécastaings, Santiago Herrero, Elizabeth A. Hillard, Patrick Rosa, Lorenzo Poggini, Matteo Mannini, Miguel Cortijo, Mathieu Gonidec

**Affiliations:** † 129863 Univ. Bordeaux, CNRS, Bordeaux INP, ICMCB, UMR 5026, F-33600 Pessac, France; ‡ Department of Chemistry ‘‘Ugo Schiff’’ (DICUS), University of Florence & INSTM Research Unit of Florence, Sesto Fiorentino 50019, Italy; § Univ. Bordeaux, CNRS, Bordeaux INP, ISM, UMR 5255, F-33405 Talence, France; || Univ. Bordeaux, CNRS, CRPP, UMR 5031, F-33600 Pessac, France; # Department of Inorganic Chemistry, Faculty of Chemical Sciences, 16734 Complutense University of Madrid, Avda. Complutense s/n, E-28040 Madrid, Spain; †† Knowledge Technology Institute, 16734 Complutense University of Madrid, Campus de Somosaguas, Pozuelo de Alarcón, Madrid E-28223, Spain; ‡‡ Istituto di Chimica dei Composti Organo-Metallici (ICCOM-CNR), Via Madonna del Piano 10, 50019 Sesto Fiorentino, Italy

## Abstract

The use of coordination compounds provides a promising strategy to unravel the factors governing chirality-induced spin selectivity (CISS), particularly the contribution of spin–orbit coupling (SOC) to its magnitude. Here, we report the formation of self-assembled monolayers on gold substrates of a chiral paramagnetic Ru_2_^5+^ paddlewheel complex, [Ru_2_(NCS)­{μ-(*S*)- or (*R*)-pycsa}_2_(μ-OAc)_2_] (pycsa = *N*-(pyridin-2-yl)-10-camphorsulfonamidate), denoted as (*S*)- or (*R*)-**RuNCS**. The incorporation of camphor groups confers point chirality to the molecule and causes a slight twisting around the Ru_2_ unit, introducing also a helical chirality, as evidenced by single-crystal X-ray diffraction and dichroic electronic absorption. The complex features an axial ligand bearing a terminal sulfur atom that serves as a robust anchoring site to the metallic surface. The resulting SAMs are homogeneous, stable, and exhibit very reproducible electrical characteristics. Magnetic-conductive atomic force microscopy measurements on enantiopure Ni/Au–**RuNCS** junctions reveal remarkable spin polarizations reaching values from 60 to 80%, which is particularly noteworthy considering the monolayer nature of the film and the modest helicity of the paddlewheel motif.

## Graphical abstract

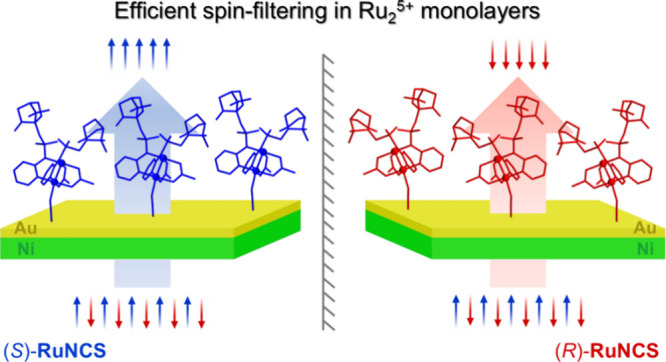

## Introduction

During the past decade, the chirality-induced spin selectivity (CISS) effect has aroused great interest among physicists and chemists and has been measured in several chiral systems. However, fundamental understanding about the factors affecting the spin polarization (SP) induced by CISS remains limited.^1^ This lack of knowledge is due to both the poor availability of comparable experimental data sets and the significant discrepancies between theory and experimental results. For example, theoretical predictions^2^ show that spin–orbit coupling (SOC) plays a key role in CISS, however, these studies fail to predict the scale of the effect by various orders of magnitude.^2^ To address this gap between theory and experiments, the development of new platforms for the study of the CISS effect is necessary to gain knowledge about the key factors that affect spin filtering in chiral molecules.

Coordination chemistry can lead to a wider understanding of this phenomenon, as it allows the introduction of versatile molecular systems, which can be easily tuned in terms of their electronic and three-dimensional structure. However, coordination compounds have rarely been exploited for research into the CISS effect. In the first such study, Cardona-Serra and co-workers reported spin polarization in two helical lanthanide-based metallopeptides.^3^ Later, the spin-dependent transport of two different amino acid–based SAMs were tested, where the biomolecules acted as both anchoring group and ligands for Cu­(II) ions.^4^ Moreover, two recent works include spin-dependent transport experiments in single crystals of Cu­(II)^5^ and Co­(II)-phenylalanine complexes.^6^ Besides molecular systems, other examples of extended coordination networks that have been investigated by the CISS community include covalent organic frameworks,^7^ metal–organic frameworks^8^ or chiral supramolecular assemblies.^9^

To further explore the potential of coordination chemistry for the study of the CISS effect, we focused on the paddlewheel motif. This geometry is particularly versatile, as chirality can be introduced by different means.^10^ These include the use of chiral ligands, the generation of a helical axis due to ligand steric hindrance, or axial chirality arising from the arrangement of the donor atoms. Among the family of paddlewheel complexes, diruthenium compounds have garnered significant attention due to their rich magnetic and redox properties.^11,12^ Structurally, these complexes consist of a bimetallic Ru–Ru core bridged by four equatorial ligands, with the coordination sphere completed by monodentate ligands occupying none, one or both of the axial positions. The most stable and frequently encountered species feature two ruthenium centers respectively in the +3 and the +2 formal oxidation state. However, a strong electronic delocalization within the ruthenium-based orbitals results in highly stable metal–metal bonds with a formal bond order of 2.5. Due to this bonding, each ruthenium center effectively adopts an average valence of 2.5, and the complexes are consequently considered to contain Ru_2_^5+^ units. The electronic structure of Ru_2_^5+^ complexes typically features a molecular orbital energy spectrum in which the π* and δ* orbitals are nearly degenerate, leading to species with three unpaired electrons.^13^

The stability, unique electronic structure and chiral versatility of the Ru_2_^5+^ moiety make it an attractive platform for investigating the CISS effect. In this context, our recent study of the racemic helical complex [Ru_2_(NCS)­(μ-ap)_4_] (ap = 2-anilinopyridinate) has shown that Ru_2_^5+^ paddlewheel complexes can be effectively anchored to gold surfaces through NCS^–^ axial ligands, forming stable and electronically robust self-assembled monolayers (SAMs).^14^ Building on this work, we here aim extend this architecture to enantiopure systems by using Ru_2_^5+^ units supported by chiral ligands, thus providing further experimental insight into spin-selective electron transport through chiral metal-based frameworks.

## Results and Discussion

### Synthesis and Characterization of the Compounds

The synthetic methodology is shown in Figure 1a. First, two acetate ligands in [Ru_2_Cl­(μ-OAc)_4_][Bibr ref15] were replaced by one or the other enantiomer of *N*-(pyridin-2-yl)-10-camphorsulfonamide ((*S* or *R*)-**Hpycsa**),^16^ yielding [Ru_2_Cl­{μ-(*S*)- or (*R*)-pycsa}_2_(μ-OAc)_2_], (*S* or *R*)-**RuCl** hereafter. This was accomplished by sonication of a suspension of [Ru_2_Cl­(μ-OAc)_4_] and the corresponding enantiomer of **Hpycsa** in ethanol/Et_3_N. A complex/ligand ratio of 1:1.9 was employed for the reaction, since the remaining [Ru_2_Cl­(μ-OAc)_4_] can be easily removed by filtration. The use of different complex/ligand proportions – ligand excess (1:8), ligand deficiency (1:1) and stoichiometric proportions (1:2) – invariably led to the exclusive formation of the disubstituted (*S* or *R*)-**RuCl**. In order to provide an anchoring motif for the construction of self-assembled monolayers (SAMs), the axial chloride ligand in (*S* or *R*)-**RuCl** was quantitatively replaced by NCS^–^, using KNCS, and giving rise to the complex [Ru_2_(NCS)­{μ-(*S*)- or (*R*)-pycsa}_2_(μ-OAc)_2_], (*S* or *R*)-**RuNCS**. Purity of all samples were confirmed by satisfactory elemental analyses and thin layer chromatography.

**Figure 1:**
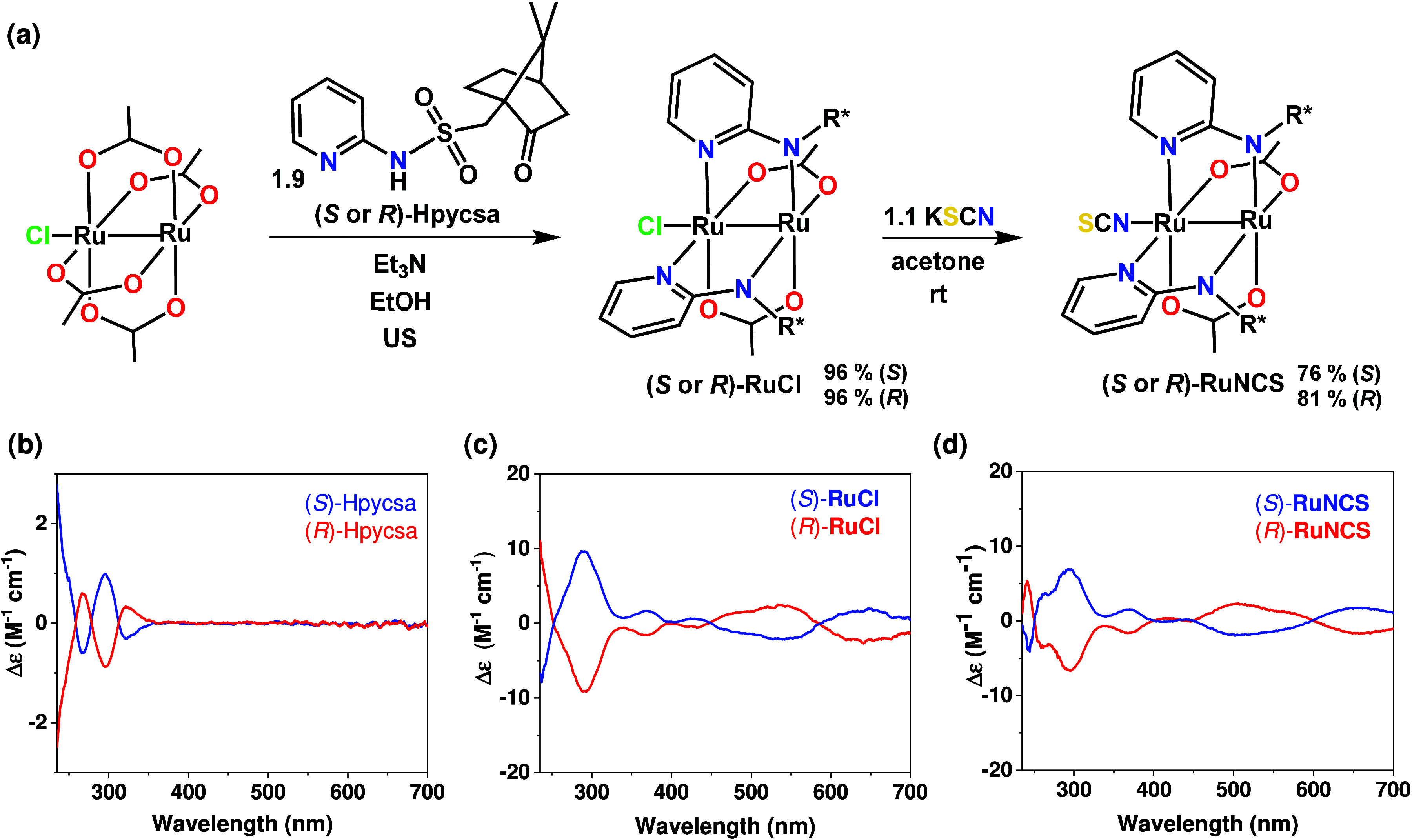
(a) Synthesis route followed for the preparation of both enantiomers of **RuCl** and **RuNCS**. R* = 10-camphorsulfonyl; circular dichroism spectra of dichloromethane solutions of: (b) (*S*)-Hpycsa (blue) and (*R*)-Hpycsa (red); (c) (*S*)-**RuCl** (blue) and (*R*)-**RuCl** (red); (d) (*S*)-**RuNCS** (blue) and (*R*)-**RuNCS** (red).

The compounds in the bulk phase were analyzed by elemental analysis, mass spectrometry (MS), as well as infrared (IR), ultraviolet–visible (UV–vis) and circular dichroism (CD) spectroscopies. Using the positive electrospray ionization (ESI^+^) mode, the mass spectra show a main fragment which corresponds to the loss of the axial ligand, [M - X]^+^ (X = Cl^–^, NCS^–^) (see Figure S2). Indeed, for these kind of complexes, the molecular peak is typically absent due to the labile character of the axial ligands.^17–20^ However, for the isothiocyanate derivative **RuNCS**, a significant signal (17%) at *m*/*z* = 994.3 can be assigned to [M]^+^, suggesting a stronger binding of the axial ligand compared to the chloride analogue. The IR spectra of the complexes **RuCl** and **RuNCS** are shown in Figure S3. The two complexes display almost identical spectra, except for the appearance of a strong band at ∼ 2035 cm^–1^ for **RuNCS**, characteristic of the CN stretching vibration of the axial NCS^–^ ligand. The CN stretching energies are typically higher in S-bonded complexes (∼2100 cm^–1^) than in N-bonded complexes (near or below 2050 cm^–1^).^21^ Thus, the IR data suggest a coordination to the metal center through the N atom in **RuNCS**.^14,21,22^

The electronic spectra of dichloromethane solutions of **Hpycsa** and of the derived **RuCl** and **RuNCS** complexes are reported in Figures S4 and S5. The most relevant absorption bands found for each compound are listed in the SI with a tentative assignment based on experimental and theoretical data found in the literature for similar compounds,^23^ and on time-dependent density functional theory calculations (TD-DFT) performed on the **RuNCS** complex (see Tables S1 and S2 and Figures S6–S8). Regarding the free ligand, three absorption maxima can be observed in the ultraviolet region at 249, 273, and 316 nm, which are assigned to n → π* and π → π* transitions. Likewise, various absorptions are observed in the ultraviolet region for the complexes: the two highest in energy, at 246 and 277 nm, can be assigned to intraligand π → π* transitions, while the other one, at 375 nm (**RuCl**) and 399 nm (**RuNCS**) could be given by π­(N/O/aryl) → σ*­(Ru_2_/axial) transitions. In the visible range, one shoulder at 432 nm for **RuCl** and two significant absorptions in the visible region are observed for the two compounds, at 471 and 498 nm and at 552 and 551 nm, which are assigned to a combination of ligand-to-metal charge transfer and Ru_2_ → Ru_2_ transitions (see Table S2). Lastly, a shoulder is observed in both spectra at 633 and 640 nm, ascribed to other Ru_2_ → Ru_2_ transitions.^23^

The chiroptical properties of dichloromethane solutions of both enantiomers of **Hpycsa** and the complexes were measured in the ultraviolet–visible range (Figure 1b–d). As expected for a noncolored species, the free ligand only exhibits CD in the ultraviolet region, with three signals at 267, 295, and 321 nm. For the **RuCl** and **RuNCS** complexes, CD bands are also observed in that region, given by the presence of the chiral ligand, in addition to a new signal around 370 nm. Notably, both complexes exhibit CD signals in the visible range. A subtle signal appears around 420 nm, along with more intense ones in the 480–550 nm range, consistent with ligand-to-metal charge transfer bands (see Table S2).^23^ Lastly, another signal appears for both pairs around 650 nm, ascribed to metal–metal transitions. The exact mechanism giving rise to these chiroptical responses is not of obvious steric origin, since both structures have a near-eclipsed conformation in the solid state (*vide infra*). From the TD-DFT results, one can deduce that the bands in the 480–550 nm range result mostly from a chirality transfer from the pycsa ligands to the metal centers. In contrast, the dichroic signals above 600 nm likely arise from the slight twisting of the structure around the metal–metal bond axis and may thus be more linked to the particular electronic configuration and delocalized metal–metal bond.

The crystal structures of (*S*)-**RuCl**·0.5THF, (*R*)-**RuCl**·0.5THF, (*S*)-**RuNCS**·0.5toluene·solvent, and (*R*)-**RuNCS**·0.5toluene·solvent (where the solvent consists of disordered cyclohexane molecules) were successfully solved and refined in the Sohncke orthorhombic space group *P*2_1_2_1_2_1_. A summary of the crystal structures and refinement data, along with a view of the asymmetric units, and the most relevant bond distances and angles are shown in Tables S3–S10 and Figures S9–S12in the Supporting Information. The refined Flack parameters for these structures are statistically indistinguishable from zero, confirming that the absolute configuration determined by resonant diffraction matches the configuration of the starting materials in all cases. Both **RuCl**·0.5THF, and **RuNCS**·0.5toluene·solvent display a characteristic paddlewheel structure with two acetate and two pycsa ligands positioned equatorially in a *cis* configuration. It should be noted that **RuNCS**·0.5toluene·solvent contains two paddlewheel motifs within the asymmetric unit. In all four structures, the pyridinic N atoms of both pycsa ligands are coordinated to the same metal atom, the one bonded to the axial ligand. As a result, its coordination environment is octahedral, whereas the other ruthenium atom presents a square pyramidal geometry. Figure 2 shows both enantiomers of the **RuNCS** molecule.

**Figure 2:**
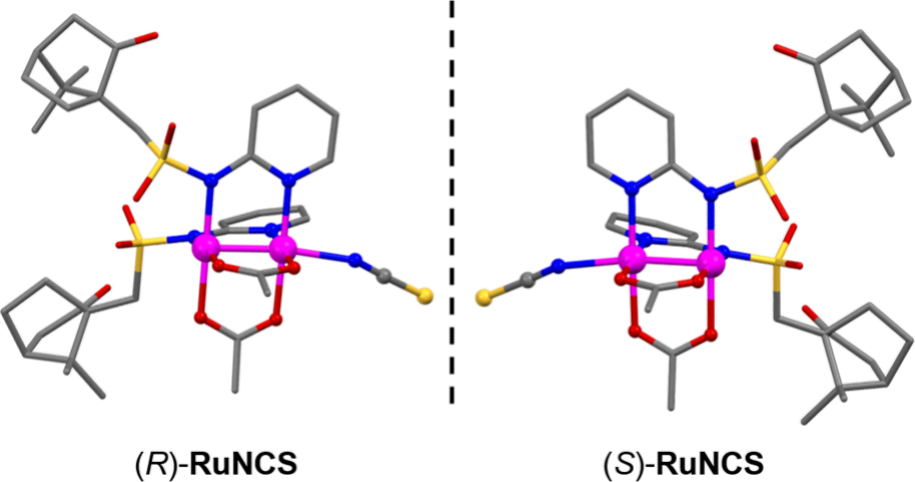
Simplified view of the structure of both enantiomers of **RuNCS**·0.5toluene·solvent. Ru atoms are shown in pink, N in blue, S in yellow, O in red and C in gray. H atoms and solvent molecules have been omitted for clarity.

The Ru–Ru distances in all structures are similar, ranging from 2.285(1) to 2.2906(6) Å, consistent with a metal–metal bond order of 2.5 in Ru_2_^5+^ species. The chloride ligand and the nitrogen atoms of the isothiocyanate ligands are nearly collinear with the ruthenium atoms, with Ru–Ru–Cl angles of 170.51(2)° and 170.50(4)° in **RuCl**·0.5THF and Ru–Ru–N angles in the 171.9(3)° to 172.9(3)° range in **RuNCS**·0.5toluene·solvent. The isothiocyanate ligands in the **RuNCS**·0.5toluene·solvent complexes are tilted with respect to the metal–metal axis, displaying Ru–N–C angles in the range of 148(1)° to 150.5(5)°. Regarding the intra- and intermolecular interactions observed in the crystal both **RuCl**·0.5THF and **RuNCS**·0.5toluene·solvent exhibit similar key inter- and intramolecular C–H···O interactions. The oxygen atoms from the acetate ligands interact with a hydrogen atom of a camphor group from the adjacent diruthenium unit, as well as with a hydrogen atom from a camphor group of the same unit. Additionally, the oxygen atoms of the sulfonyl groups of the pycsa ligands establish interactions with the camphor groups within the same diruthenium unit, as well as with the hydrogen atoms of the camphor moiety and acetate groups from neighboring units (see Figures S13 and S14).

When considering the diruthenium paddlewheel motif of the **RuNCS** compound, it initially appears to be essentially eclipsed (see Figure S15), but a more careful examination reveals a consistent twisting of the ligands introducing a very slight helical conformation around the Ru–Ru axis (see Figure S16). This twisting is more pronounced in one of the two molecules that are present in the unit cell, however, they both feature the same handedness (see Table S11). Following standard nomenclature, (*R*)-**RuNCS** shows thus right-handed helicity (Δ-**RuNCS**) and (*S*)-**RuNCS** shows left-handed helicity (Λ-**RuNCS**). While the origin of this twisting is not obvious, its surprising stability is attested by the presence and persistence of the dichroic signals in solution reported above, which were observed in the visible range where mainly metal–metal or ligand-to-metal transitions are observed.

Along with the structural characterization, variable temperature and field magnetization measurements (Figures S17 and S18) were carried out on (*S*)-**RuCl**, and (*S*)-**RuNCS** to verify the *S* = 3/2 spin state arising from the Ru_2_^5+^ oxidation state. The experimental values obtained for χ_M_ *T* at 300 K in both cases are close to the expected spin-only value for a quadruplet state (*g* = 2, 23.6 × 10^–6^ m^3^ K mol^–^^1^) that arises from a σ^2^π^4^δ^2^(δ*π*)^3^ configuration (23.48 and 23.63 × 10^–6^ m^3^ K mol^–1^, respectively). Lowering the temperature results in a decrease of these values, mainly caused by zero-field splitting (ZFS). Variable temperature and variable field magnetization data were simultaneously fitted with the PHI software^24^ considering a *S* = 3/2 state, with purely axial for (*S*)-**RuCl** or rhombic zero-field splitting for (*S*)-**RuNCS**. Adding an intermolecular magnetic exchange (*zJ*) term included as a perturbation on the molecular field improved the fit in both cases. The ZFS *D*(*E*) values found were 62.9 K for (*S*)-**RuCl** and 78.7(−17.9) K for (*S*)-**RuNCS**. Table S12 shows the parameters obtained from the best fits to the data.

Cyclic voltammograms in dichloromethane solutions of (*S*)-Hpycsa, (*S*)-**RuCl** and (*S*)-**RuNCS** were recorded. Within the anodic and cathodic limits of dichloromethane, no redox processes were observed for Hpycsa. For the complexes, a clear quasi-reversible one-electron reduction assigned to the redox pair Ru_2_^5+/4+^ is observed (see Figure S19). The exchange of the chloride axial ligand in **RuCl** by an isothiocyanate group results in an anodic shift of the reduction potential (see Table S13). The π-donor character of the chloride ligand results in a higher electron-density on the Ru_2_^5+^ core compared to the NCS^–^ analogue, making **RuCl** harder to reduce. In addition, a less relevant reduction process was registered, which might be given by the reduction of other minor Ru_2_^5+^ species that can be formed in solution.^25^ This is a known behavior for these compounds and is ascribed to an exchange between the axial ligands and the anions from the supporting electrolyte.^26^

### Preparation of SAMs and Surface Characterization

The protocol followed for the preparation of Au-**RuNCS** was previously reported by some of us for another Ru_2_^5+^ complex bearing an NCS^–^ axial ligand.^14^ Samples were obtained by incubating substrates in a ∼ 0.5 mM solution of **RuNCS** in toluene for 3.5 h at room temperature (Scheme S1). In parallel, samples were produced using a **RuCl** solution to confirm the crucial role of the NCS^–^ axial ligand in forming robust chemically grafted monolayers.

CD experiments were performed in order to exclude ligand reorganization or decomposition during incubation (see Figure S20). The CD spectra of fresh toluene solutions were recorded at times 0 and 3.5 h at room temperature and no significant changes were observed. Additionally, fresh toluene solutions were refluxed for 1 h and the CD was checked after cooling to room temperature. Once again, no differences were found, assuring the integrity of the molecules prior to the anchoring to the surface.

A detailed surface analysis was carried out for the SAMs of the *S* enantiomer using atomic force microscopy (AFM), time-of-flight secondary ion mass spectrometry (ToF-SIMS), polarization modulation infrared reflection absorption spectroscopy (PM-IRRAS), and X-ray photoelectron spectroscopy (XPS). Standard thermally evaporated Cr/Au substrates were employed for ToF-SIMS and PM-IRRAS characterizations. Hydrogen flame annealed gold on mica substrates were employed when preparing samples for XPS. Template-stripped gold (Au^TS^) substrates were used for AFM, magnetic-conducitve AFM (mc-AFM) control tests and EGaIn charge transport experiments, while Au/Ni/Ti template-stripped multilayer (AuNi^TS^) substrates were used to prepare samples for mc-AFM to evaluate CISS.

AFM topography of the Au^TS^-**RuNCS** sample (Figure 3) showed a very smooth surface with an RMS roughness of 3.3 Å, comparable to that of the bare template-stripped gold substrates (2.9 Å, Figure S21).^14^ Consistent with previous observations for a similar complex,^14^ the sharp gold grain boundaries (see Figure S21) were softened by the monolayer (see Figure 3), and the most prominent features are pinholes due to defects in the underlying gold film. ToF-SIMS was employed to verify the presence of the Ru complexes on the Au surface. Both negative and positive secondary ion mass spectra were recorded for Au-**RuNCS** and the control **RuCl** samples. In the negative ion mode, no relevant information was obtained (Figures S22 and S23). On the contrary, the positive mode spectra confirmed the presence of the **RuNCS** complex on the substrates (Figure 4), while the control **RuCl** sample featured only weak signals (Figure S24).

**Figure 3:**
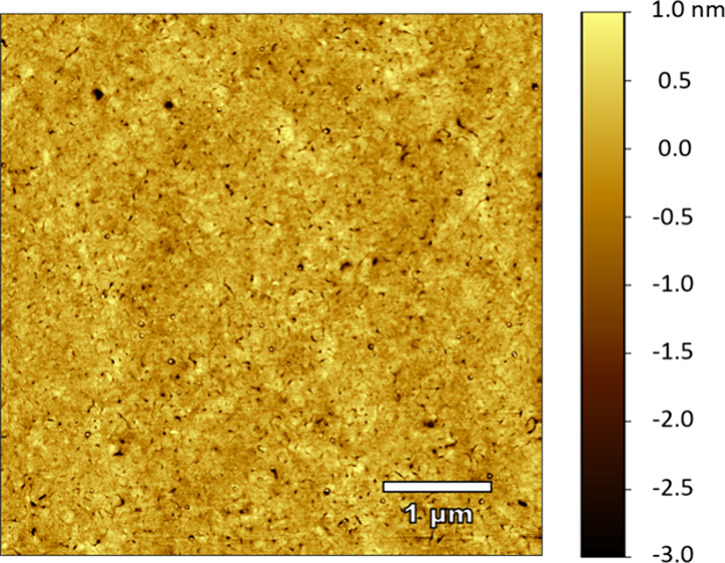
Tapping-mode AFM topography image of the Au^TS^-**RuNCS** sample.

**Figure 4:**
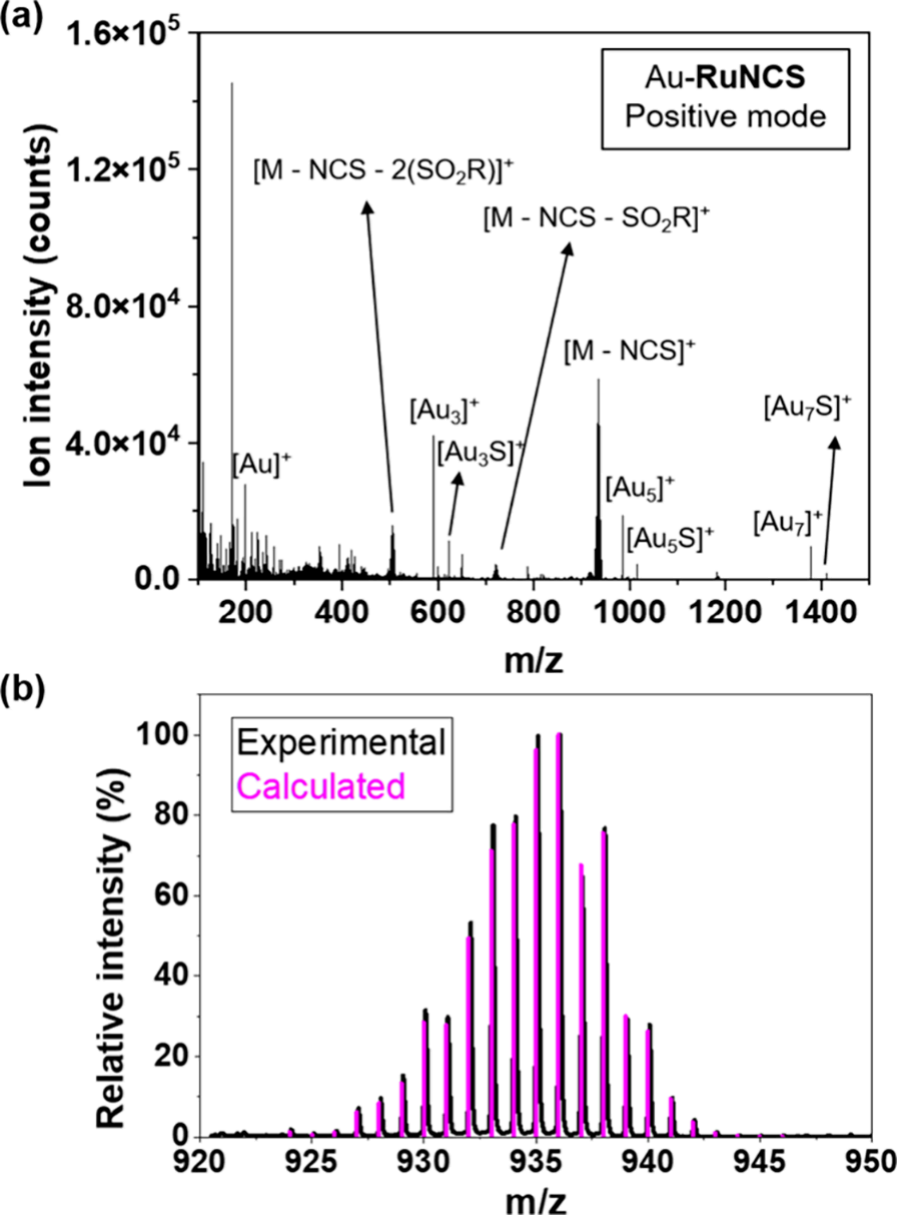
(a) ToF-SIMS spectrum of ions derived from Au^TS^-**RuNCS** in positive mode; (b) enlargement of the [M - NCS]^+^ peak. R = 10-camphorsulfonyl.

Three main fragments were observed for Au-**RuNCS:** [M - X]^+^, [M - X - SO_2_R]^+^ and [M - X - 2­(SO_2_R)]^+^ (where X = axial ligand, R = 10-camphoryl). Moreover, different Au clusters were observed (Figure 4). Notably, various [Au_*x*_S]^+^ fragments were detected, indicative of sulfur–gold bond formation. As expected for these compounds, the most intense peak corresponds to the [M - X]^+^ fragment, arising from the loss of the axial ligand during the measurement. Figure S25 compares the ion intensities of the [M - X]^+^ fragment normalized with respect to the intensity of the Au_5_^+^ ions for the **RuCl** and **RuNCS** samples, revealing an intensity about 40 times greater for the NCS^–^ derivative. This pronounced difference is consistent with the assumption that **RuCl**, which lacks a terminal group capable of strong chemisorption onto gold, is merely physisorbed, while **RuNCS** forms a chemically bound monolayer. A two-dimensional mapping analysis of the most relevant ions observed in the spectra was performed to obtain information about the surface pattern in a point-by-point manner. These 2D ToF-SIMS images taken for Au-**RuNCS** are shown on Figure S26 and demonstrate the homogeneity of the SAMs.

The PM-IRRAS spectrum recorded for a freshly prepared Au-**RuNCS** monolayer (Figure 5) displayed essentially the same bands as the bulk sample, apart from the clear shift of the – N=C—S asymmetric stretching band. This shift, from 2035 to 2090 cm^–^^1^ is consistent with the anchoring of the complex to the gold substrate via the sulfur atom, suggesting the formation of a SCN bridge between the Au surface and the Ru atom (Ru-NCS-Au).^21^

**Figure 5:**
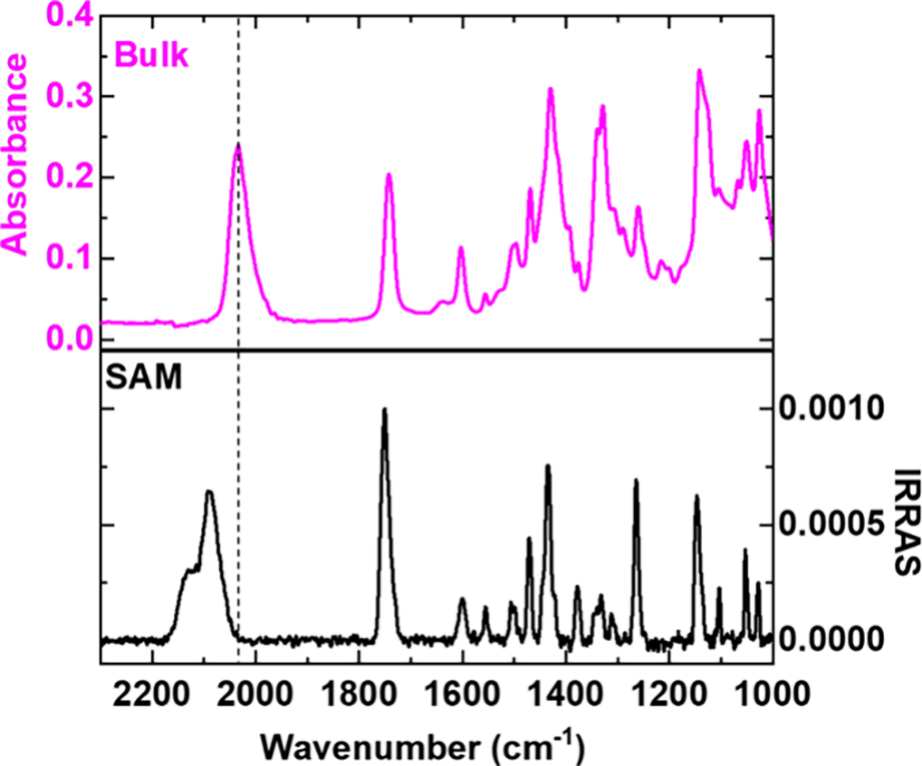
Comparison of the IRRAS spectrum of Au-**RuNCS** (black) with the IR spectrum of **RuNCS** (pink).

To confirm the correct assembly of the SAMs, XPS measurements were performed on a Au-**RuNCS** monolayer and compared to a bulk reference sample, prepared by dropcasting the same solution used for the preparation of the **RuNCS** SAMs on a gold surface. The S2*p*, N1*s* and Ru3*d* regions were analyzed to perform a semiquantitative analysis of the chemical composition of the Au-**RuNCS** SAM. Figure 6 shows a comparison between the spectra of the SAM and the bulk material for the three regions investigated.

**Figure 6:**
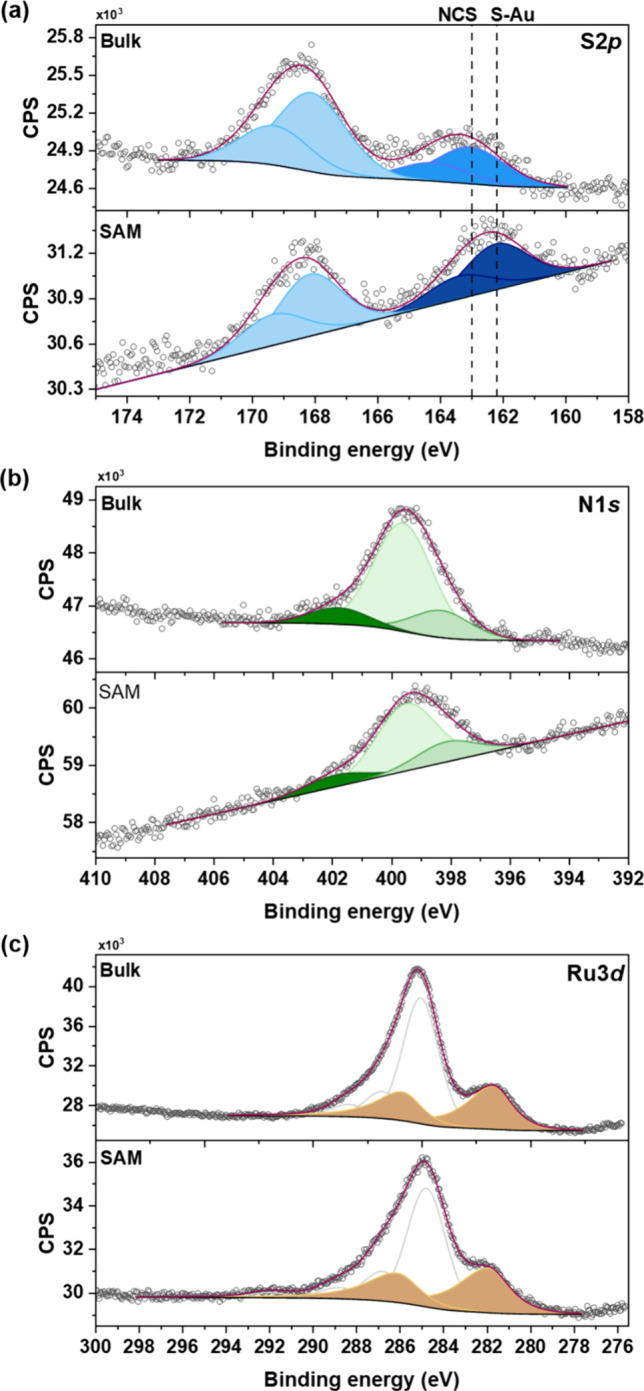
(a) S2*p*, (b) N1*s*, and (c) Ru3*d* XPS region acquired on (*S*)-**RuNCS** bulk sample (top) and on Au-(*S*)-**RuNCS** SAM (bottom). The dashed lines in panel (a) highlight the position of the component attributed to NCS species and to the formation of S–Au bonds.

The analysis of the S2*p* region confirms covalent binding of the complex to the gold substrate through the terminal S atom (Figure 6a). Indeed, the bulk sample shows two components at 168.1 and 163.0 eV, which are ascribed to the SO_2_ groups of the chiral ligand and the NCS^–^ axial ligand, respectively, with the expected 2:1 ratio. In the monolayer, the latter component is shifted toward lower binding energies (162.2 eV), which demonstrates Au–S bond formation in accordance with reported binding energy values for Au-SCN.^27^ The deviation from the theoretical 2:1 ratio between the two components suggests partial molecular degradation, likely due to the interaction between the SO_2_ groups of the ligand and the metallic surface. By exploiting the SO_2_ component as an internal standard for intact molecules, the contribution from the S–Au component arising from degraded species was estimated to be approximately 13%.

In the N1*s* region (Figure 6b), components corresponding to the nitrogen donor atoms of the pycsa and NCS^–^ ligands are observed at 399.6 and 398.3 eV, respectively.^28^ An additional feature observed at higher binding energy is assigned to a *shakeup* peak of the pyridinic ring. Comparison between the spectra of the bulk sample and the SAM reveals that the same chemical environments of the nitrogen atoms are retained after monolayer formation. The same components can be observed in the monolayer sample (399.5 and 398.2 eV), with only minor baseline differences due to the proximity to the Au*4d*_*3/2*_ signal of the substrate.

The Ru3*d*_5/2_ components are present in both the bulk (281.7 eV) and the SAM (281.9 eV) spectra with the corresponding spin–orbit coupling (Figure 6c). Notwithstanding the partial overlap with the C1*s* contribution, the strong similarity between the two samples further confirms that the Ru environment is not perturbed by covalent anchoring of the complex on the gold surface.^29^

Semiquantitative elemental analyses were carried out for the S2*p*, N1*s*, and Ru3*d* spectral regions to estimate the stoichiometry in both the bulk and surface-bound samples (Table S14). To properly evaluate the stoichiometry of the monolayer sample, the contribution to the sulfur component provided by the decomposed molecules has been excluded. Considering the experimental uncertainties of the XPS technique, the elemental analyses support that the molecules of **RuNCS** deposited on the surface remain mainly intact.

### Charge Transport Measurements

To evaluate the electrical robustness of the SAMs, we studied the charge transport through Au^TS^-**RuNCS** monolayers by forming large-area molecular junctions with an eutectic gallium–indium (EGaIn) top electrode.^30,31^ The measurements were performed in the ± 0.5 V range across freshly prepared Au^TS^-**RuNCS** monolayers. Among 14 independent junctions, only two exhibited short circuits. As a control, Au^TS^-**RuCl**//Ga_2_O_3_/EGaIn junctions were also tested under identical conditions and showed a much higher shorting rate (7/10), in agreement with the low stability and low surface coverage one could expect from such thoroughly washed physisorbed samples.

Although the Au^TS^-**RuNCS** monolayers displayed good overall stability, occasional shorts were also observed. The average current density at +0.50 V was log|J|/(A·cm^–2^) = −0.60 ± 0.47 (N = 482) (Figure S27). This value is slightly lower than that previously reported for an analogous diruthenium complex,^14^ although the standard deviation is slightly larger. Altogether, these characteristics demonstrate good electrical robustness of the Au^TS^-**RuNCS** SAMs, an important prerequisite for the reliable assessment of the CISS effect by mc-AFM.

### mc-AFM Measurements: Study of the CISS Effect

To evaluate the magnitude of the CISS effect across **RuNCS** monolayers, charge transport measurements were performed using mc-AFM. In this technique, the current flowing through the monolayer is measured by applying a bias voltage between a magnetized Ni/Au substrate and the grounded, conductive Pt coated AFM tip. Enantiopure monolayers of Ni/Au-(*S*)-**RuNCS** or Ni/Au-(*R*)-**RuNCS** were assembled on ferromagnetic substrates following the procedure described in the Supporting Information. Hundreds of individual *I/V* curves were collected to extract statistically relevant data over the sample surface (Figures S28).

Thick Ni films formally present an in-plane magnetic anisotropy. However, MFM experiments have shown that in such systems, there is still a net nonzero finite remanent out-of-plane magnetization^32^ which, in principle, makes it possible to measure CISS in remanence. Figure 7a,b shows averaged *I*/*V* curves for Ni/Au-(*S*)-**RuNCS** and Ni/Au-(*R*)-**RuNCS**, measured in remanence after magnetizing the Ni layer with a 0.5 T magnet oriented either parallel or antiparallel to the surface normal. The mc-AFM *I/V* curves exhibit similar profiles to the average *J*/*V* curve measured using large-area EGaIn junctions (Figure S29). Most importantly, these results show that the current intensity depends on the out-of-plane orientation of the remanent magnetization of the ferromagnetic substrate for both enantiomers. Notably, this dependence reverses between the two chiral forms, indicating that each enantiomer favors the transport of charge carriers of a specific spin state. This behavior confirms the occurrence of a spin-selective charge transport arising from the handedness of the **RuNCS** monolayers.

**Figure 7:**
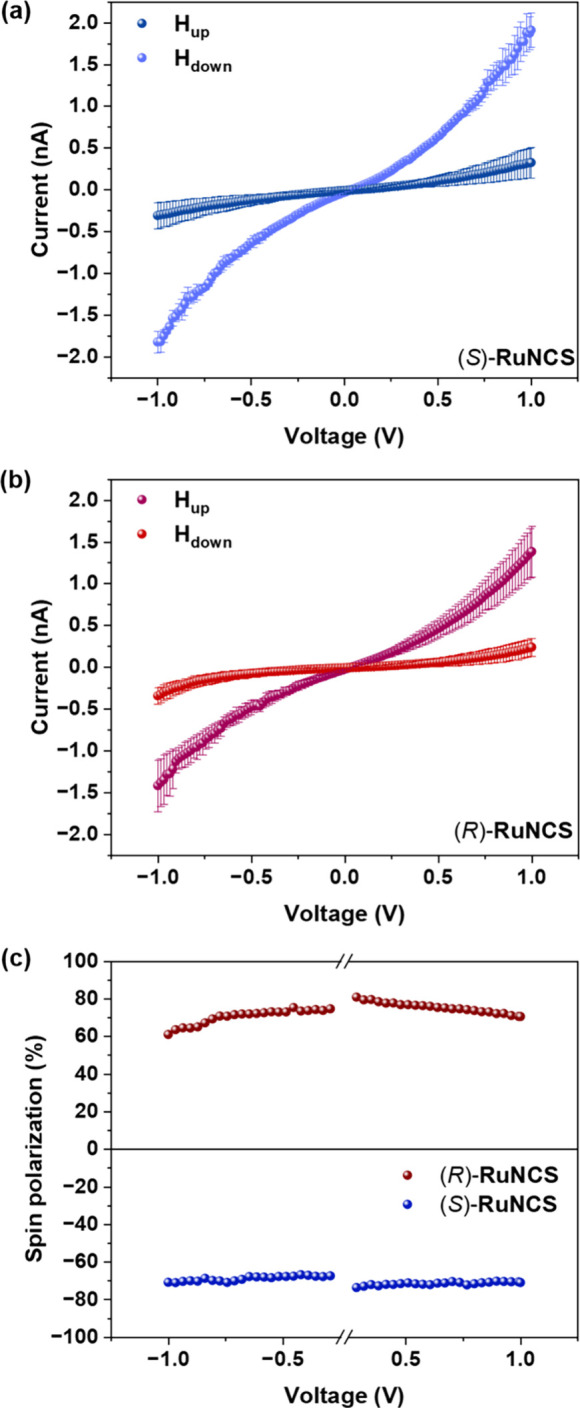
*I*/*V* curves acquired on (a) Ni/Au-(*S*)-**RuNCS** and on (b) Ni/Au-(*R*)-**RuNCS** applying either positive or negative magnetic field of 0.5 T and measuring in remanent conditions. The error bars correspond to the standard error $\sigma /\sqrt{n}$ where σ is the standard deviation and *n* is the number of measurements. (c) Spin polarization percentage extracted from the corresponding *I*/*V* curve for SAMs of both enantiomers at room temperature.

There are two main phenomena that could account for such an observation: the CISS effect and the electrical magnetochiral anisotropy (eMChA) effect.^33,34^ Although these two phenomena can exhibit similar magnitudes, they differ significantly in their voltage and magnetic field dependences. In particular, the spin polarization (SP) induced by the CISS effect is expected to be independent of the applied voltage, whereas the magnetoconductance arising from eMChA is proportional to the applied voltage.

As a result, for a pure CISS effect, the *I*/*V* curves measured for opposite substrate magnetizations cross at zero bias and yield a voltage-independent SP. In contrast, for a pure eMChA contribution, the difference between the two *I/V* characteristics scales linearly with voltage and therefore vanishes at zero bias, where the two curves are tangent. This results in a magnetoconductance that increases linearly with the applied voltage and exhibits no net effect at zero bias.

In some cases, as recently demonstrated for helicene molecular junctions,^34^ both effects can coexist, yielding a linear dependence of the magnetoconductance with the applied voltage (due to eMChA) with a net nonzero offset at zero bias (due to CISS). The voltage-independent response observed here (Figure 7c) is, therefore, consistent with a pure CISS contribution and indicates the absence of any significant electrical magnetochiral anisotropy.

The same measurements were performed on diamagnetic Au^TS^ substrates to confirm that the observed effect is neither ascribable to eMChA (which can be observed even with diamagnetic electrodes) nor to the paramagnetic nature of the compound. Following the same procedure adopted for the measurements described above, the samples were first exposed to a magnetic field, after which the *I/V* curves were acquired (Figure S30). The resulting average curves show no dependence on the magnetic field orientation, confirming that, as expected, the SP cannot be observed in the absence of a magnetized ferromagnetic Ni layer acting as a spin analyzer (Figure S31). This finding unambiguously corroborates the occurrence of a pure CISS effect in these systems.

To further validate the observations reported in Figure 7, additional measurements were also performed on AuNi^TS^(*R*/*S*)-**RuNCS** at room-temperature under a static magnetic field of about ± 0.2 T applied perpendicular to the sample surface generated by a NdFeB permanent magnet placed underneath the substrate during the *I*/*V* curves acquisition (see Figure S32). This experimental configuration allows measurements to be obtained with the ferromagnetic substrate maintained at its magnetic saturation throughout the acquisition. As shown in Figure S33a,b, the curves are comparable to those obtained under remanent magnetization conditions, suggesting that the CISS response shows little dependence on substrate magnetization within the investigated range.

A similar comparison can be made by estimating the SP for all samples using eq S1. For substrates in remanent magnetization, the (*R*)-**RuNCS** monolayer reaches a SP between 60 and 80%, depending on the applied voltage, with lower voltage values being more affected by the experimental uncertainty on the measured currents. As expected, the (*S*)-**RuNCS** monolayer shows a comparable SP magnitude with opposite sign (Figure 7c). The same analysis performed on diamagnetic Au^TS^ electrodes yields negligible SP values for both enantiomers (see Figure S31c), further confirming that the observed spin selectivity originates from the interplay between molecular chirality and the ferromagnetic substrate. Finally, curves obtained under an applied magnetic field reveal a slightly lower SP, between 50% and 80%, with a response that depends on the enantiomer, similarly to what is observed in remanent conditions, but the SP values are more voltage-dependent (Figure S33c). When measured in remanence the junctions appeared to be more stable. The observation of the CISS effect is likely possible in this configuration due to the partial spin polarization of the ferromagnetic layer featuring a surface domain structure widely described in literature via MFM experiments.^32^

It is worth noting that all of the SP values reported here are particularly high compared to those reported elsewhere for other d-block coordination compounds with analogous setups. Among the few examples reported in the literature, the spin-dependent transport in amino acid Cu­(II) compounds only showed a maximum SP of ∼ 5%, due to additional transport pathways provided by the two different amino acidic structures.^4^ In single crystals of Cu­(II)^5^ and Co­(II)-phenylalanine complexes,^6^ a SP of up to 68% and 45% was found, respectively. Unlike molecular systems, some extended coordination networks such as covalent organic frameworks^7^ and metal–organic frameworks^8^ have been reported to behave as essentially ideal spin filters (SP > 90%).

Given the modest helicoidal character of **RuNCS**, such a high SP is somewhat unexpected. Comparable SP values (up to 80%) to those reported here for **RuNCS**, were observed in extended nanographenes exhibiting helical chirality and strong chiroptical responses.^35^ Indeed usually such high SP values are mainly associated with helical chirality. As previously shown for DNA-based monolayers,^36^ systems with helical conformations such as double-stranded DNA induce a greater SP than those with only point chirality such as single-stranded DNA, which essentially shows no SP even with multiple point chiral centers.^37^ The large SP observed here is therefore noteworthy for a molecule showing mainly point chiral centers, which are seen to induce only a slight helicoidal twisting of the paddlewheel bonds.

This leads us to speculate that the nature of the metal centers contributes to the high SP, either due to their high SOC or to the paramagnetic nature of the system. Regarding paramagnetism, the influence of unpaired electrons in the system remains yet unclear for the CISS community. To the best of our knowledge, there are only two comparative studies in the literature, and these are contradictory. On one hand, studies on organic molecules showed negligible differences in SP between a radical cation and the corresponding neutral diamagnetic species.^38,39^ On the other hand, a pronounced dependence on the magnetic properties of the metal center was reported for metallopeptides, where the substitution of a diamagnetic Y^3+^ ion by a paramagnetic Tb^3+^ ion increased the SP significantly from 50% to 70%.^3^

It is remarkable that the main difference between these two studies is the magnitude of the change of SOC when moving from the diamagnetic compound to the paramagnetic analogue. In the case of the organic system, the SOC is very small in both species. However, in the case of the metallopeptides the electronic configuration of the metal ions changes from [Kr]­4d^0^ (Y^3+^) to a [Xe]­4f^8^ (Tb^3+^). This difference in electronic configuration results in a large change in the magnitude of the SOC, with that of the Y^3+^ being much smaller than that of the lanthanide. Based on these two reports, one can therefore speculate that the high SP values in **RuNCS** might arise mostly because of the high SOC of the ruthenium ions. However, the influence of the paramagnetic nature of the compound on the CISS response cannot be fully discarded. This will be the subject of more systematic studies in the near future.

## Conclusions

Enantiopure diruthenium paddlewheel complexes, (*S*)- and (*R*)-**RuNCS**, were synthesized and shown to form smooth, robust self-assembled monolayers on gold through covalent anchoring of the isothiocyanate axial ligand. Comprehensive surface analyses confirmed that the Ru_2_^5+^ cores mostly retained their structural integrity and electronic structure upon surface immobilization. Charge transport studies demonstrated stable junction behavior, while mc-AFM measurements revealed clear and significant enantio-complementary spin filtering with SP values reaching up to 80% with a structural combination of point chirality and a slight twisting introducing some helical chirality around the Ru–Ru paddlewheel axis. Furthermore, an intriguing lack of correlation between the CISS effect yield and the magnetization strength of the substrate was observed within the investigated range. We believe that the significant spin polarization is likely linked to the presence of the Ru_2_^5+^ unit. Further comparative studies in this family of compounds are under way to establish whether this is due to the strong SOC constant of the 4d metal ions or to the presence of unpaired electrons.

## Supplementary Material



## Data Availability

A selection of the experimental data was deposited on the French national open data repository recherche.data.gouv.fr with the following DOI: https://doi.org/10.57745/084AMP.

## Notes

Materials and methods section, and the data for mass spectrometry, IR spectroscopy, electronic absorption spectroscopy, SCXRD, magnetic measurements, cyclic voltammetry, AFM, ToF-SIMS, XPS, EGaIn junctions, and mc-AFM (PDF)

## ¶

CIC nanoGUNE, Tolosa Hiribidea 76, 20018, Donostia/San Sebastián, Spain
