# Supplementary material for: Efficient Chirality-Induced Spin Selectivity in Self-Assembled Monolayers of Ru2 5 + Paddlewheel Complexes
Source: J Am Chem Soc. 2026 Jun 27;148(27):28283–92. doi: 10.1021/jacs.6c02811 (PMC13383615; doi:10.1021/jacs.6c02811)
Supplement: Supplementary file 1 [file ja6c02811_si_001.pdf]

Efficient Chirality-Induced Spin Selectivity in Self-Assembled Monolayers of Ru_2^{5+} Paddlewheel Complexes

Isabel Coloma,[†] Niccolò Giaconi,[‡,∦] Francesco Parmeggiani,[‡] Thierry Buffeteau,[§] Gilles Pécastaings,[‖] Santiago Herrero,[#,††] Elizabeth A. Hillard,[†] Patrick Rosa,[†] Lorenzo Poggini,[‡,‡‡,*] Matteo Mannini,[‡] Miguel Cortijo,[#,*] and Mathieu Gonidec[†,*]

[†]Univ. Bordeaux, CNRS, Bordeaux INP, ICMCB, UMR 5026, F-33600 Pessac, France

[‡]Department of Chemistry "Ugo Schiff" (DICUS) & INSTM Research Unit, University of Florence, Sesto Fiorentino 50019, Italy

[§]Univ. Bordeaux, CNRS, Bordeaux INP, ISM, UMR 5255, F-33405 Talence, France

[‖]Univ. Bordeaux, CNRS, CRPP, UMR 5031, F-33600 Pessac, France

[#]Department of Inorganic Chemistry, Faculty of Chemical Sciences, Complutense University of Madrid, Avda. Complutense s/n, E-28040 Madrid, Spain

[††]Knowledge Technology Institute, Complutense University of Madrid, Campus de Somosaguas, E-28223 Pozuelo de Alarcón, Madrid, Spain

[‡‡]Istituto di Chimica dei Composti Organo-Metallici (ICCOM-CNR), Via Madonna del Piano 10, 50019, Sesto Fiorentino, Italy

Corespondence: Miguel Cortijo (miguelcortijomontes@ucm.es), Mathieu Gonidec (mathieu.gonidec@icmcb.cnrs.fr), Lorenzo Poggini (lorenzo.poggini@cnr.it)

TABLE OF CONTENTS

Experimental part 3

General materials and methods 3

Single-crystal X-ray diffraction data collection and structure refinement 3

Synthesis of the complexes 4

Substrate preparation 5

Preparation of SAMs of diruthenium complexes on Au, Au^{TS} or Ni/Au 6

Surface characterization 6

Density Functional Theory (DFT) calculations 7

Charge transport measurements 7

Magnetic-conductive atomic force microscopy (mc-AFM) 8

Characterization of the complexes 9

Mass spectrometry 9

Infrared spectroscopy 10

Electronic spectroscopy 11

Magnetic measurements 29

Complex stability 32

Surface characterization 33

Atomic Force Microscopy 33

Time-of-flight secondary ion mass spectrometry (ToF-SIMS) 34

X-ray photoelectron spectroscopy (XPS) 37

EGaIn and magnetic-conductive atomic force microscopy (mc-AFM) measurements 38

References 42

Experimental part

General materials and methods

All solvents and reactants were obtained from commercial sources and used as received without further purification. Elemental analyses were performed by the Microanalytical Service of the Complutense University of Madrid. Mass spectrometric analyses of bulk samples were carried out employing an LQC Fleet Ion Trap spectrometer (Thermo Fisher) in direct injection mode with a 1:1 mixture of dichloromethane/methanol. FT-IR spectra were recorded with a PerkinElmer Spectrum 100 instrument equipped with a universal ATR accessory. Electronic spectra of $\sim 10^{-4}$ M dichloromethane solutions were recorded at room temperature employing a Cary 5G spectrometer. Electronic circular dichroism spectra were acquired using a JASCO-1500 circular dichroism spectrometer equipped with a Peltier thermoelectric temperature controller. Variable-temperature (VT) and variable-field (VH) magnetization measurements were performed using a Quantum Design MPMX-7XL SQUID magnetometer using 10.71 mg (VT) and 3 mg (VH) of (*S*)-**RuCl**, and 9.63 mg of (*S*)-**RuNCS**. The measurements were carried out under a 0.5 T magnetic field for (*S*)-**RuCl** and under a 1.0 T magnetic field for (*S*)-**RuNCS**. The data were corrected for the diamagnetic contribution of both the sample and the sample holder. Thermogravimetric analysis was performed employing a TGA Perkin-Elmer Pyris 1 under a N_2 atmosphere by heating the sample at 5 °C/min. Cyclic voltammetry measurements were performed using a Metrohm Autolab PGSTAT204 potentiostat at a scan rate of 0.1 V s^{-1}. The experiments were conducted in a 0.05 M tetrabutylammonium perchlorate (TBAP) solution in degassed dichloromethane, employing a conventional three-electrode setup: a glassy-carbon working electrode, a Pt-wire counter electrode and a Ag/AgCl pseudo-reference electrode. Ferrocene in a 0.05 M TBAP dichloromethane solution was employed as a reference, yielding the ferrocenium/ferrocene couple at 0.56 V vs. Ag/AgCl.

Single-crystal X-ray diffraction data collection and structure refinement

Single crystals of (*S*)-**RuCl**·0.5THF and (*R*)-**RuCl**·0.5THF were measured at room temperature using a Bruker APEX-II CCD diffractometer with Mo K_α radiation (λ = 0.71073 Å) at the Institute of Condensed Matter Chemistry of Bordeaux. Data collection for (*R*)-**RuNCS**·0.5toluene·solvent (solvent = cyclohexane) was carried out at 100 K using a Rigaku Synergy-S diffractometer with Mo K_α radiation in the Autonomous University of Madrid X-ray diffraction service. The corresponding (*S*)-**RuNCS**·0.5toluene·solvent (solvent = cyclohexane) crystal structure was measured at 100 K in the X-ray Diffraction Service of Complutense University of Madrid, employing a D8 venture diffractometer with Cu K_α radiation (λ = 1.54178 Å). CCDC 2515274-2515277 contain the crystallographic data for the structures reported in this article. Structures were solved using OLEX2[1] software with SHELXT[2] using intrinsic phasing and then refined with SHELXL2018[3] using least squares minimization. All non-hydrogen atoms were refined anisotropically. Hydrogen atoms were placed in geometrically calculated positions and refined using a riding model with fixed isotropic displacement parameters. For (*S* and *R*)-**RuNCS**·0.5toluene·solvent, the asymmetric unit contains two diruthenium molecules, one toluene molecule, and severely disordered cyclohexane

molecules. The disordered solvent contribution was treated using the OLEX2 solvent mask routine with a probe radius of 1.2 Å. The resulting residual electron densities correspond to 886.5 electrons per unit cell for (*S*)-**RuNCS**·0.5toluene·solvent and 755.9 electrons for (*R*)-**RuNCS**·0.5toluene·solvent, equivalent to approximately for 1.85 and 1.57 cyclohexane molecules per chemical formula, respectively. Thermogravimetric analysis of freshly filtered (*S*)-**RuNCS**·0.5toluene·solvent (Figure S1) showed a mass loss of 15.6% between 35 °C and 200 °C, consistent with the release of 0.5 toluene molecules and 1.5 cyclohexane molecules per formula unit. The small discrepancy between the TGA and crystallographic data likely results from partial solvent loss at room temperature prior to measurement.

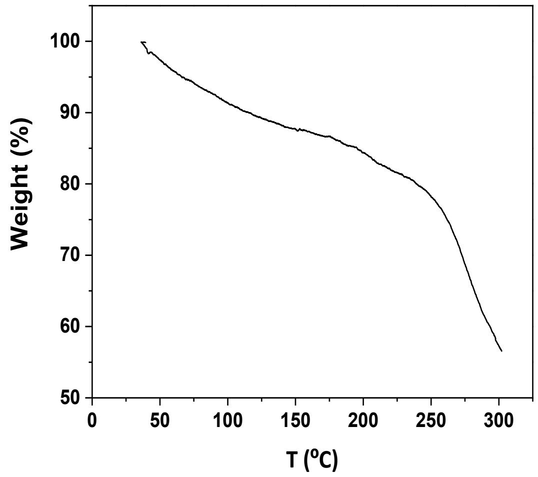

Figure S1. TGA of *(S*)-**RuNCS**·0.5toluene·solvent (solvent = 2 cyclohexane).

Synthesis of the complexes

*Cis-[Ru$_2$Cl{μ-(S or R)-pycsa}$_2$(μ-OAc)$_2$] ((S or R)-**RuCl**).* [$Ru_2Cl(\mu\text{-}OAc)_4$] (100.00 mg, 0.21 mmol), (*S* or *R*)-**Hpycsa** (120.00 mg, 0.40 mmol), Et_3N (0.15 mL, 1.08 mmol) and absolute ethanol (25 mL) were added to a Schlenk flask. The resulting suspension was sonicated for 3 h at 80 kHz. The mixture was filtered through Celite® and the solvent evaporated. The solid obtained was redissolved in 20 mL of dichloromethane and the solution was added to an extraction funnel and washed with distilled water (2 × 20 mL). The organic phase was dried over magnesium sulfate and filtered. The solvent was evaporated under vacuum yielding a red solid. Yield (*S*): 96% (190 mg). Elemental analysis (*S*) for $C_{34}H_{44}ClN_4O_{10}S_2Ru_2{\cdot}H_2O$ (988.486 gmol^{-1}): % found (theoretical); C, 41.40 (41.31); H, 4.65 (4.69); N, 5.65 (5.67); S, 6.42 (6.49). Yield (*R*): 96% (189 mg). Elemental analysis (*R*) for $C_{34}H_{44}ClN_4O_{10}S_2Ru_2{\cdot}H_2O$ (988.486 gmol^{-1}): % found (theoretical); C, 40.76 (41.31); H, 4.53 (4.69); N, 5.54 (5.67); S, 6.19 (6.49). IR: $\tilde{\nu}$ (cm^{-1}) = 3118w, 3042w, 2961m, 2892w, 1741s, 1603m, 1557w, 1504m, 1471s, 1428vs, 1394s, 1376m, 1306s, 1292m, 1248m, 1217m, 1201w, 1141vs, 1052m, 1026m, 943s, 837vs, 768s, 732s, 694vs, 685vs, 655m, 597s, 571vs. UV/Vis-NIR (CH_2Cl_2): λ_{max} (ε/ M^{-1}cm^{-1}) = ~246 sh (17000), 277 (13000), ~375 sh (1700), ~432 sh (2000), 471 (2600), 552 (3000), ~633 sh (1300) nm. CD UV/Vis (*S*) (CH_2Cl_2): λ (Δε/ M^{-1}cm^{-1}; Δε/ε) = 288 (9.71; 8.36 × 10^{-4}), 368 (1.68; 9.25 × 10^{-4}), 426 (0.63; 3.36 × 10^{-4}), 532 (-2.13; 7.72 × 10^{-4}), 642 (2.03;

1.61 × 10^{-5}) nm. CD UV/Vis (*R*) (CH_2Cl_2): λ (Δε/ $M^{-1}cm^{-1}$) = 289 (-9.21; -7.93 × 10^{-4}), 367 (-1.63; -8.97 × 10^{-4}), 427 (-0.57; -3.00 × 10^{-4}), 533 (2.50; 8.99× 10^{-4}), 642 (-2.67; -2.10 × 10^{-5}) nm. MS (ESI$^+$): m/z = 993.886 (6%), $[M + Na]^+$; 936.088 (100%), $[M - Cl]^+$. Single crystals of (*S*)-**RuCl**·0.5THF and (*R*)-**RuCl**·0.5THF were obtained by slow diffusion of cyclohexane into a THF solution of the corresponding enantiomer.

*Cis-[Ru_2(NCS){μ-(S or R)-pycsa}$_2$(μ-OAc)$_2$] ((S or R)-**RuNCS**).* A solution of KSCN (11.00 mg, 0.11 mmol) in 5 mL of acetone was added to a solution of (*S* or *R*)-**RuCl** (100.00 mg, 0.10 mmol) in 5 mL of acetone. The mixture was stirred overnight and then filtered through Celite® and the solvent evaporated. The resulting solid was dissolved in 15 mL of dichloromethane. The mixture was filtered, and the solution was washed with distilled water (2 × 15 mL) employing an extraction funnel. The organic phase was dried over magnesium sulfate and filtered. The solution was taken to dryness yielding a red-pinkish solid, which was washed with hexane (2 × 5 mL). Yield (*S*): 76% (83 mg). Elemental analysis (*S*) for $C_{35}H_{44}N_5O_{10}S_3Ru_2\cdot H_2O\cdot 0.5CH_2Cl_2$ (1053.582 gmol^{-1}): % found (theoretical); C, 40.50 (40.47); H, 4.51 (4.50); N, 6.66 (6.65); S, 9.01 (9.13). Yield (*R*): 81% (87 mg). Elemental analysis (*R*) for $C_{35}H_{44}N_5O_{10}S_3Ru_2\cdot 0.5\text{hexane}\cdot 0.25H_2O$ (1040.693 gmol^{-1}): % found (theoretical); C, 43.66 (43.86); H, 4.82 (4.99); N, 6.82 (6.73); S, 9.18 (9.24). IR: $\tilde{\nu}$ (cm^{-1}) = 3118w, 3044w, 2960m, 2923m, 2852w, 2035s, 1743s, 1640w, 1603m, 1556w, 1497m, 1470s, 1429vs, 1393s, 1376m, 1339s, 1330s, 1305m, 1290m, 1260m, 1217m, 1201w, 1142vs, 1106m, 1068m, 1052s, 1027s, 953s, 944s, 909w, 851s, 837vs, 816s, 767s, 695vs, 684vs, 657m, 598s, 571vs. UV/Vis-NIR (CH_2Cl_2): λ_{max} (ε/ $M^{-1}\cdot cm^{-1}$) = ~246 sh (22000), 277 (18000), ~399 sh (3200), 498 (7700), ~551 sh (6400), ~640 sh (1700) nm. CD UV/Vis (*S*) (CH_2Cl_2): λ (Δε/ $M^{-1}cm^{-1}$; Δε/ε) = 244 (-4.06; -2.29 × 10^{-4}), 263 (3.76; 2.68 × 10^{-4}), 293 (6.97; 6.74 × 10^{-4}), 369 (1.53; 7.75 × 10^{-4}), 416 (-0.14; -5.00 × 10^{-5}), 502 (-2.01; -3.40 × 10^{-4}), 655 (1.77; 1.23 × 10^{-3}) nm. CD UV/Vis (*R*) (CH_2Cl_2): λ (Δε/ $M^{-1}cm^{-1}$; Δε/ε) = 241 (5.42; 2.68 × 10^{-4}), 262 (-3.65; -2.36 × 10^{-4}), 295 (-6.72; -6.39 × 10^{-4}), 367 (-1.65; 6.74 × 10^{-4}), 418 (0.36; 1.00 × 10^{-4}), 506 (2.34; 3.30 × 10^{-4}), 657 (-1.74; -1.00 × 10^{-3}) nm. MS (ESI$^+$): m/z = 936.003 (100%), $[M\text{-}NCS]^+$; 994.287 (17%), $[M]^+$ Single crystals of (*S*)-**RuNCS** and (*R*)-**RuNCS** were obtained by slow diffusion of cyclohexane in a solution of the corresponding enantiomer in toluene.

Substrate preparation

Template-stripped gold substrates (Au^{TS}) were prepared following the standard procedure previously described.[4] A 200 nm-thick layer of gold was deposited on a polished silicon wafer using a Korvus Technology HEX thermal evaporator. No adhesion layer was employed, and glass chips were bonded to the gold surface using photocurable NOA 61 optical adhesive (Norland). For the preparation of conventional gold substrates, a 10 nm chromium adhesion layer was first deposited onto the silicon wafer, followed by the deposition of a 200 nm gold layer using the same thermal evaporation setup.

AuNi^{TS} substrates for magnetic conductive atomic force microscopy (mc-AFM) measurements were prepared by sequential deposition by DC magnetron sputtering using a Korvus Technology HEX setup of three consecutive layers onto a polished silicon wafer without breaking the vacuum. A 10 nm gold layer was first applied directly on silicon,

followed by a 100 nm nickel layer and a final 10 nm titanium layer as an adhesion layer and then templated stripped similarly to the Au^{TS}.

Preparation of SAMs of diruthenium complexes on Au, Au^{TS} or Ni/Au

All glassware, including syringes and vials, was thoroughly cleaned with Hellmanex III special cleaning concentrate and rinsed with milli-Q water to eliminate any potential contaminants. Scheme S1 shows the methodology employed to obtain SAMs of **RuNCS**.[5] The SAMs were prepared by immersing the gold substrates in a ~0.5 mM toluene solution of the **RuNCS** complex. After 3.5 h of incubation at room temperature, the samples were carefully rinsed with fresh toluene to remove any physisorbed material and dried under a nitrogen stream. The same procedure was followed to prepare the control samples with **RuCl**.

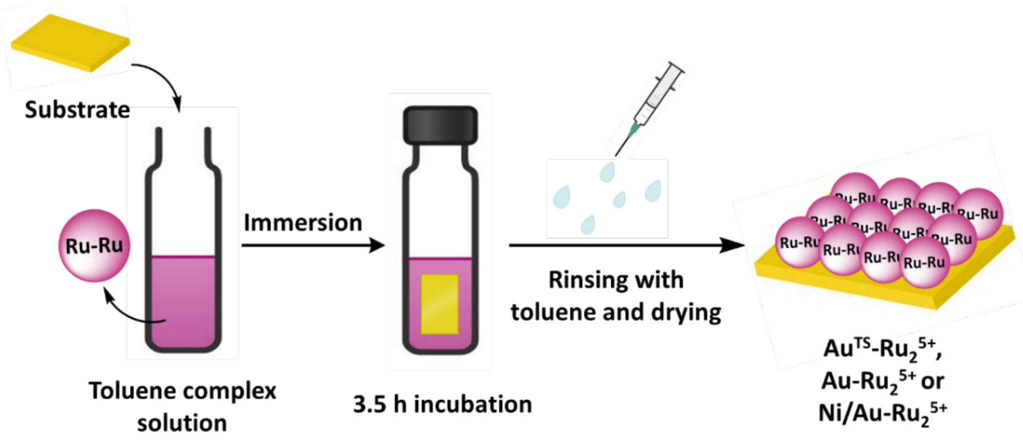

Scheme S1. Protocol followed to prepare SAMs of both enantiomers of **RuNCS**. The control samples of **RuCl** were prepared following this procedure.

Surface characterization

Atomic force microscopy (AFM): The surface morphology of the samples was characterized using a Bruker Dimension Icon AFM in tapping mode, using NCHV antimony (n)-doped Si tips, with a scan rate of 1 Hz.

Time of flight secondary ion mass spectrometry (ToF-SIMS): ToF-SIMS was employed to collect both mass spectra and two-dimensional ion distribution maps for the Au-(*S*)-**RuNCS** SAMs and the corresponding control sample (*S*)-**RuCl**. Measurements were carried out using a TOF-SIMS 5 spectrometer (IONTOF GmbH) with a bismuth liquid metal ion gun (LMIG) oriented 45° to the sample surface. The Bi^{3+} ion beam was employed at 30 kV acceleration voltage and an ion current of 0.3 pA in mass spectrometry mode. Secondary ion images were acquired over a 500 × 500 μm^2 area with a resolution of 128 × 128 pixels. The primary ion dose was maintained below 10^8 ions/cm^2.

Polarization modulation infrared reflection absorption spectroscopy (PM-IRRAS): PM-IRRAS experiments were performed on a FTIR spectrometer equipped with a PM-IRRAS optical bench, following the experimental procedure previously published.[6] The PM-IRRAS spectra were recorded at a 4 cm^{-1} spectral resolution for 5 hours of acquisition

time. The PM-IRRAS spectra were calibrated in order to be presented in IRRAS units (i.e. $1-\frac{R_p(d)}{R_p(0)}$, where $R_p(d)$ and $R_p(0)$ stand for the p-polarized reflectance of the film/substrate and bare substrate systems, respectively).[7,8]

X-ray photoelectron spectroscopy (XPS): Surface chemical composition of the Au-**RuNCS** SAMs was investigated by X-ray photoelectron spectroscopy (XPS). Measurements were conducted in an ultra-high vacuum chamber system with a base pressure of $10^{-9}/10^{-10}$ mbar. Non-monochromatic Al Kα radiation was used (hν = 1486.6 eV, VSW-A10) combined with a hemispherical electron energy analyzer (VSW-HA100 equipped with a 16-channel detector). The operating power of the Al X-ray source was 140 W (14 kV and 10 mA), maintaining the angle between the analyzer axis and an X-ray source fixed at 54.5° and photoelectrons were collected normally to the sample surface. The spectrum was acquired in fixed analyzer transmission (FAT) mode (pass energy of 44 eV) and the XPS spectra calibration was conducted by setting the Au $4f_{7/2}$ peak at 84.0 eV.[9] The fitting analyses were performed using CasaXPS software introducing mixed Lorentzian and Gaussian contributions for each component. The background was fitted using the linear or the Shirley method. The data were compared with the bulk reference sample **RuNCS** formed by drop-casting a 2 mM solution of the complex. Semiquantitative analyses of N, S and Ru was carried out using the cross-section values reported in the literature.[10]

Density Functional Theory (DFT) calculations

DFT calculations were performed using the Gaussian 09 program.[11] The geometry was optimized at the CAM-B3LYP/Def2TZVP level using an implicit polarizable continuum model for the dichloromethane solvent. Time-Dependent DFT (TD-DFT) calculations were then performed at the same level of theory to predict the CD spectrum of **RuNCS** and the results were used to generate the Natural Transition Orbitals (NTOs). Those were finally plotted at an isovalue of 0.07, for both the starting and final states, for transitions exhibiting a rotatory strength R(velocity) with an absolute value greater or equal to 3.

Charge transport measurements

Large-area junctions were formed on fresh Ru_2^{5+}-monolayers (Au^{TS}-**RuNCS**) using EGaIn junctions.[12,13] Template-stripped gold was used as bottom electrode, while selected conical tips of Ga_2O_3/EGaIn without asperities were used as top-electrodes. A syringe was loaded with Ga_2O_3/EGaIn and the tips were fabricated by contact of a drop of liquid metal with a clean sacrificial gold substrate. Tips were observed by optical microscopy while approaching the substrate to verify the soft deformation of the tip apex over the SAM, forming the desired contacts. For data collection, a slight modification of standard procedures was followed.[14] Each tip was used to form a single Au^{TS}-**RuNCS**//Ga_2O_3/EGaIn junction recording 20 J/V cycles at ±0.5 V starting at 0 V in 0.05 V steps.

Magnetic-conductive atomic force microscopy (mc-AFM)

The mc-AFM experiments were carried out for enantiopure Ni/Au-**RuNCS** SAMs using an SPM solver P47 pro system (NT-MDT Spectrum Instrument) equipped with a fully diamagnetic UHV SMENA scanning head. The current measurements were performed at room temperature. All the substrates were magnetized using a permanent magnet that generated a magnetic field of ± 0.5 T normal to the surface. Additionally, for control experiments a customized base has been developed embedding below the sample a 0.5T NdFeB permanent magnet whose orientation can be reversed manually to perform the mc-AFM control experiments under an external static magnetic field that has been evaluated at 0.22 T on the sample surface using a Hall probe sensor. By convention, H_{up} is defined when the magnetic field points out of the Au surface, whereas H_{down} corresponds to a magnetic field directed into the Au surface. Voltage ramps between ±1.0 V were applied with a Pt-coated tip (HQ:NSC18, µmasch) in contact mode applying an 8-10 nN force. Several contacts were performed across the SAMs and 100 I/V cycles were recorded for each contact. Spin polarization percentage was calculated employing Equation S1:

$$SP\ (\%) = \frac{I_{up} - I_{down}}{I_{up} + I_{down}} \times 100 \qquad \text{Equation } S1$$

where I_{up} and I_{down} refer to the intensities of the current measured when the magnetization of the nickel layer is pointing towards the molecular deposit or away from it, respectively.

The raw mc-AFM *I/V* data were processed using a custom Python script implementing a four-stage automated filtering pipeline applied globally across all junction files. In the first step, an RMS bandpass filter removed curves falling outside the 5th–95th percentile of the global RMS distribution, thereby excluding both low-signal traces (dead contacts) and abnormally high-current events (short circuits). In the second step, a relative total variation (TV) filter, calculated as TV/RMS, was applied to quantify high-frequency noise. The noisiest 3% of the remaining curves, identified as those exceeding the 97th percentile of the global TV/RMS distribution, were discarded. In the third step, a normalized median absolute deviation (NMAD) filter was used to identify shape outliers by comparing each curve to its corresponding per-junction median reference; curves with NMAD values above the 97th percentile of the global distribution were rejected, thus removing residual anomalous traces not captured by the total variation-based criterion. Importantly, all filtering thresholds were determined from the pooled distributions across all junctions rather than on a per-junction basis, preventing locally noisy datasets from biasing the selection criteria. Only junctions retaining at least 70% of their initial curves after automated filtering were considered for further analysis. Finally, an interactive quality-control step allowed visual inspection and, where necessary, exclusion of entire junction datasets prior to the calculation of the grand weighted average, in which each junction was weighted according to the number of surviving curves.

Characterization of the complexes

Mass spectrometry

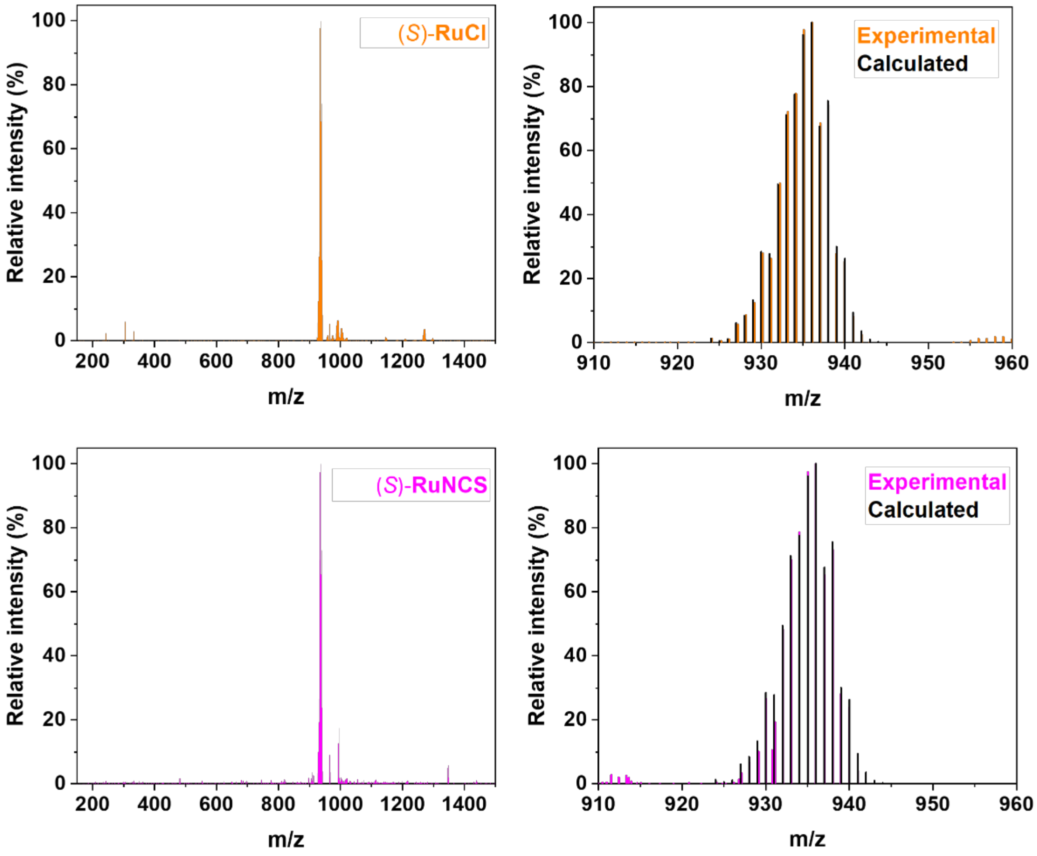

Figure S2. ESI$^+$ spectra (left) and enlargement of the $[M - X]^+$ base peak and the calculated isotopic pattern (black) (right) of (*S*)-**RuCl** (orange) and (*S*)-**RuNCS** (pink).

Infrared spectroscopy

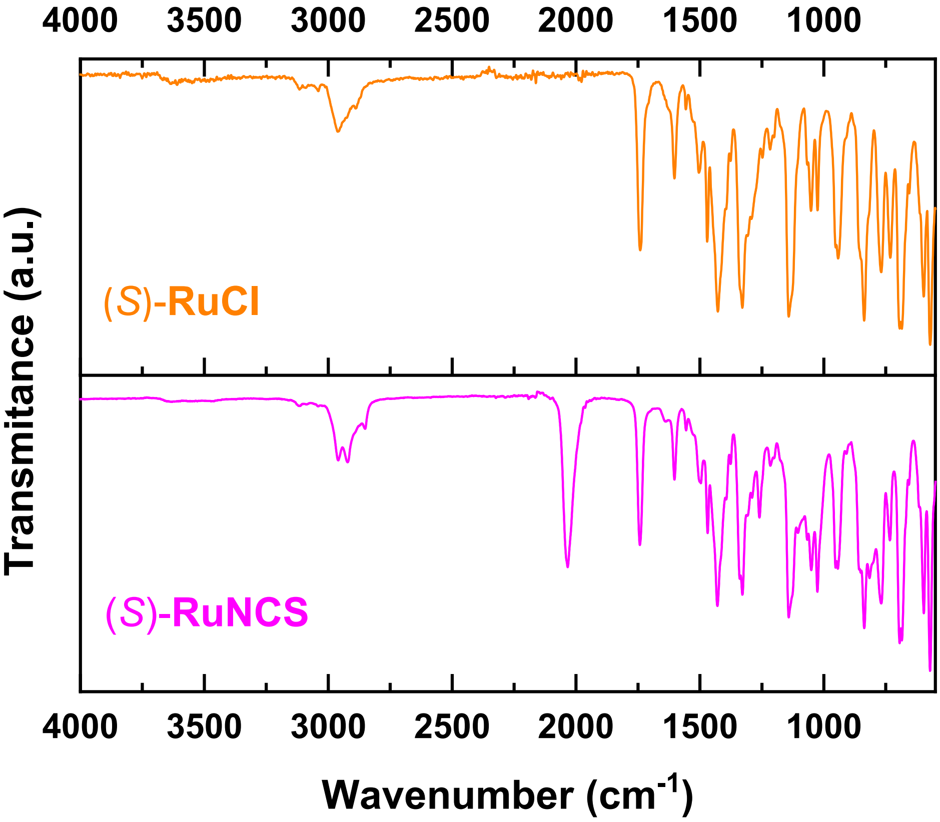

Figure S3. Infrared spectra of (*S*)-**RuCl** (orange) and (*S*)-**RuNCS** (pink).

Electronic spectroscopy

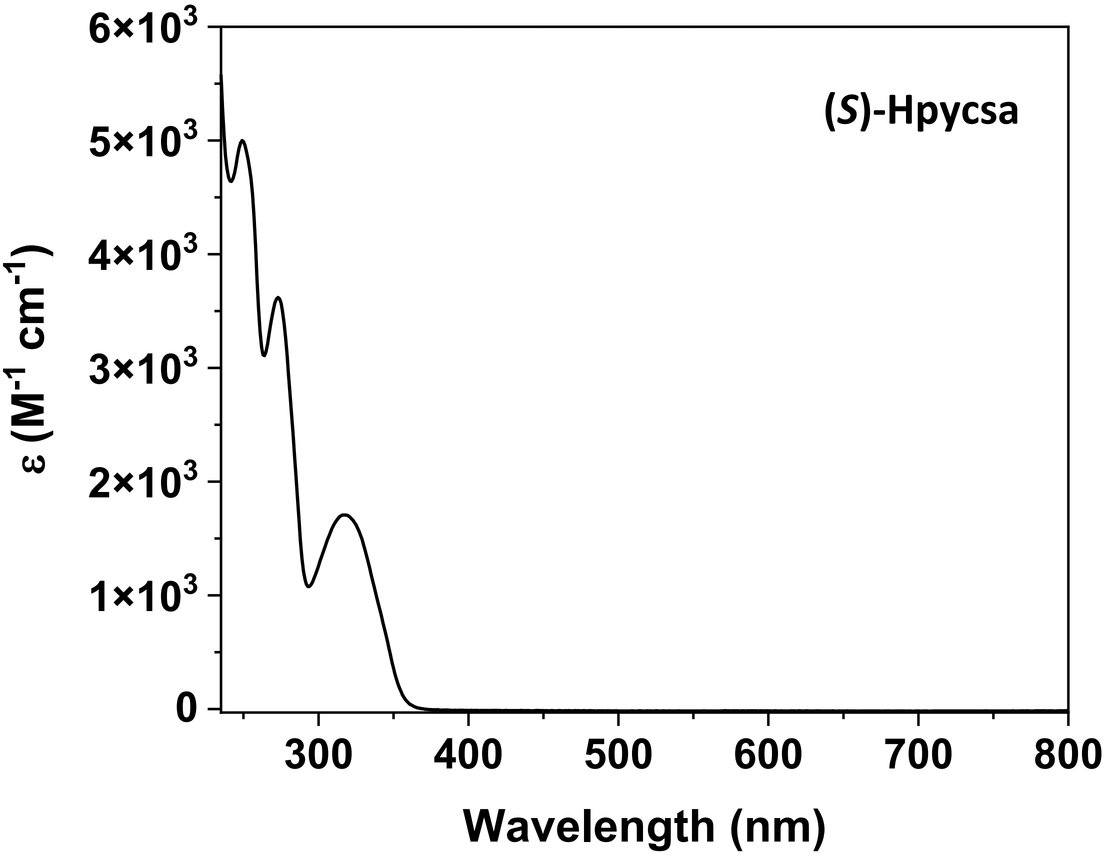

Figure S4. Electronic spectrum of a dichloromethane solution of (*S*)-Hpycsa.

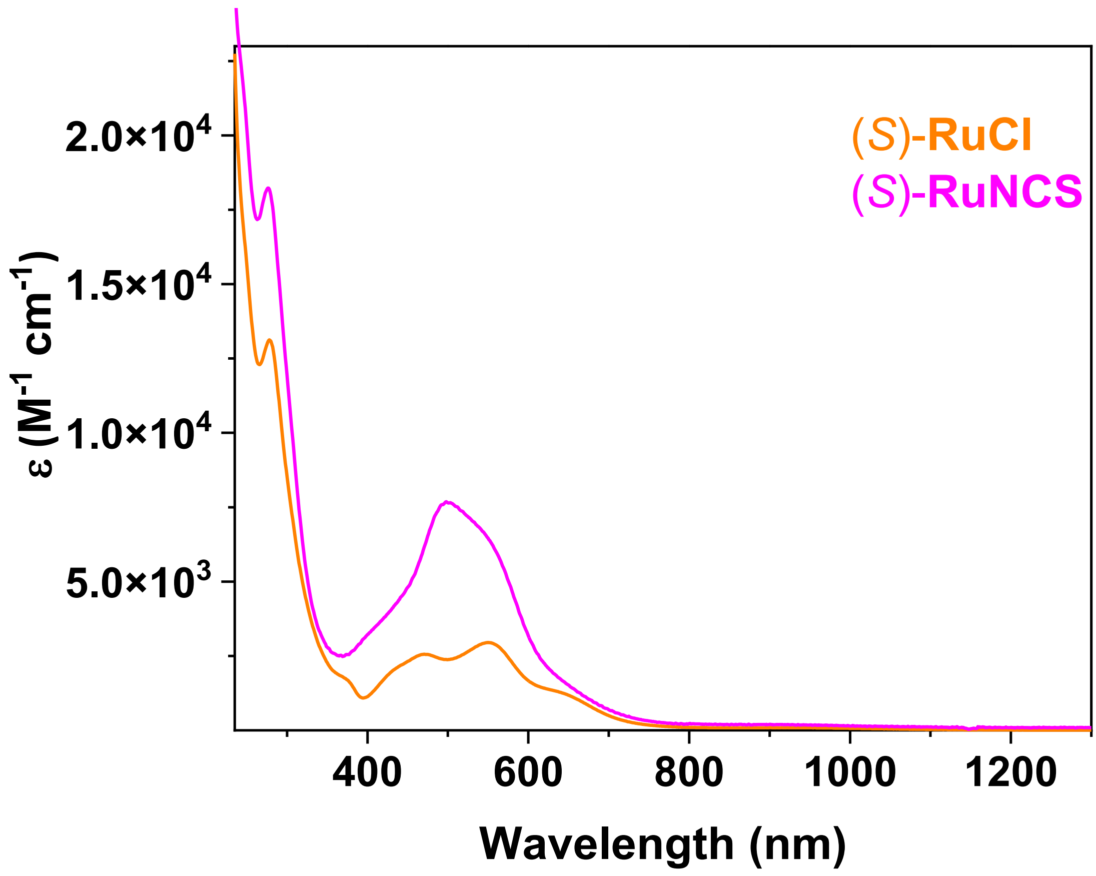

Figure S5. Electronic spectra of dichloromethane solutions of (*S*)-**RuCl** (orange) and (*S*)-**RuNCS** (pink).

Table S1. TD-DFT calculated transitions for **RuNCS** in dichloromethane.

**Transition**	**Wavelength (nm)**	**Oscillator Strength**	**Rotatory strength (10^{-40}esu^{2}cm^2)**
1	1225.1	0.000	-12.31
2	1112.3	0.000	6.77
3	1029.6	0.000	1.07
4	996.3	0.000	-0.95
5	919.8	0.000	-0.38
6	726.9	0.001	21.60
7	690.5	0.000	-3.39
8	639.3	0.024	-16.00
9	625.8	0.005	33.61
10	613.1	0.014	-13.47
11	501.2	0.001	-7.32
12	469.6	0.053	-2.99
13	442.7	0.063	-17.46
14	422.4	0.006	7.48
15	418.1	0.003	1.07
16	413.9	0.000	-0.82
17	410.3	0.005	0.25
18	407.8	0.002	-1.53
19	404.3	0.005	-0.03
20	390.5	0.000	-0.24
21	386.4	0.008	-4.22

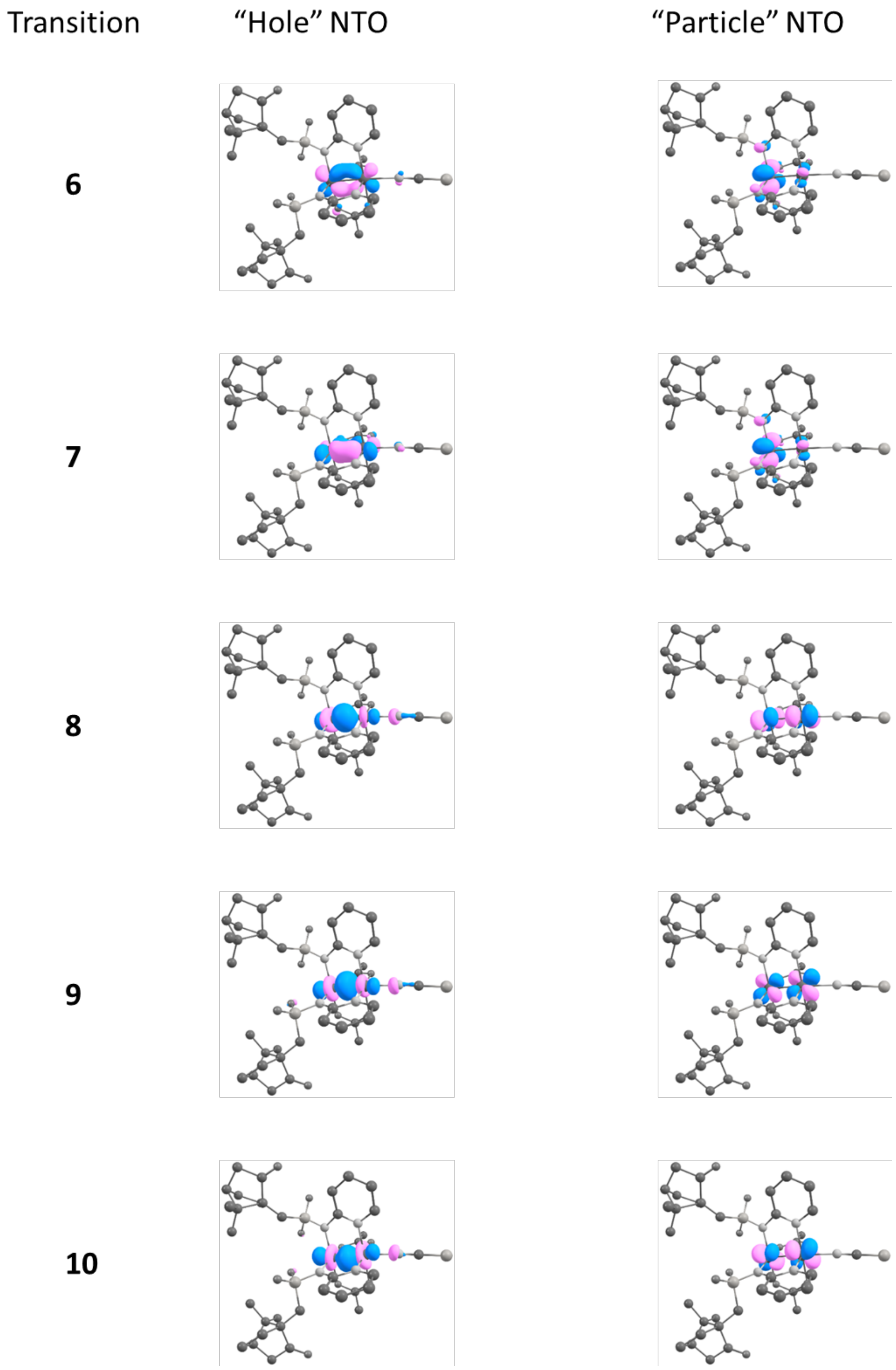

Figure S6. Natural transition orbitals obtained by TD-DFT calculations for transitions 6-10 for **RuNCS** in a dichloromethane polarizable continuum solvent model.

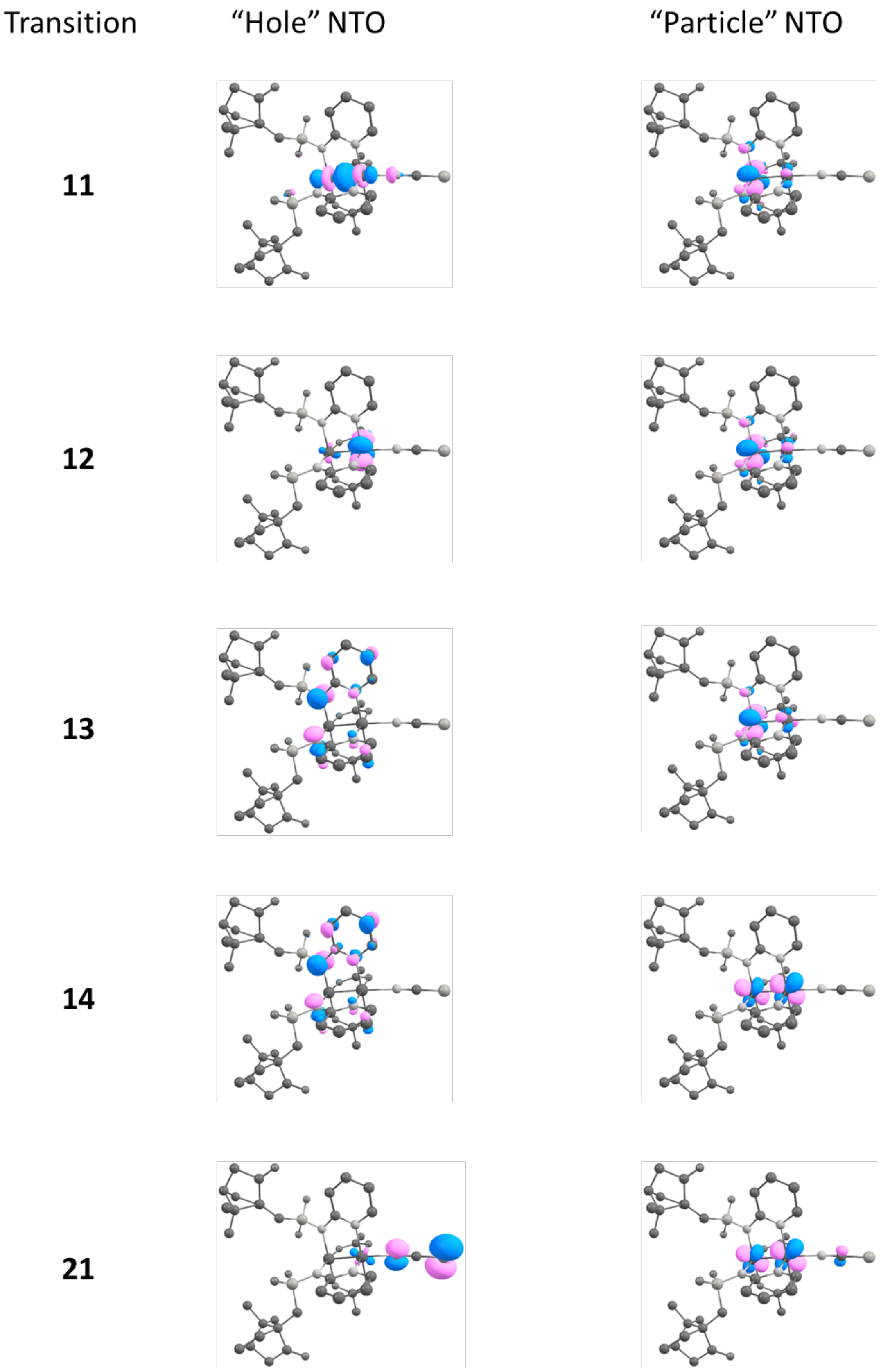

Figure S7. Natural transition orbitals obtained by TD-DFT calculations for transitions 11-14 and 21 for **RuNCS** in a dichloromethane polarizable continuum solvent model.

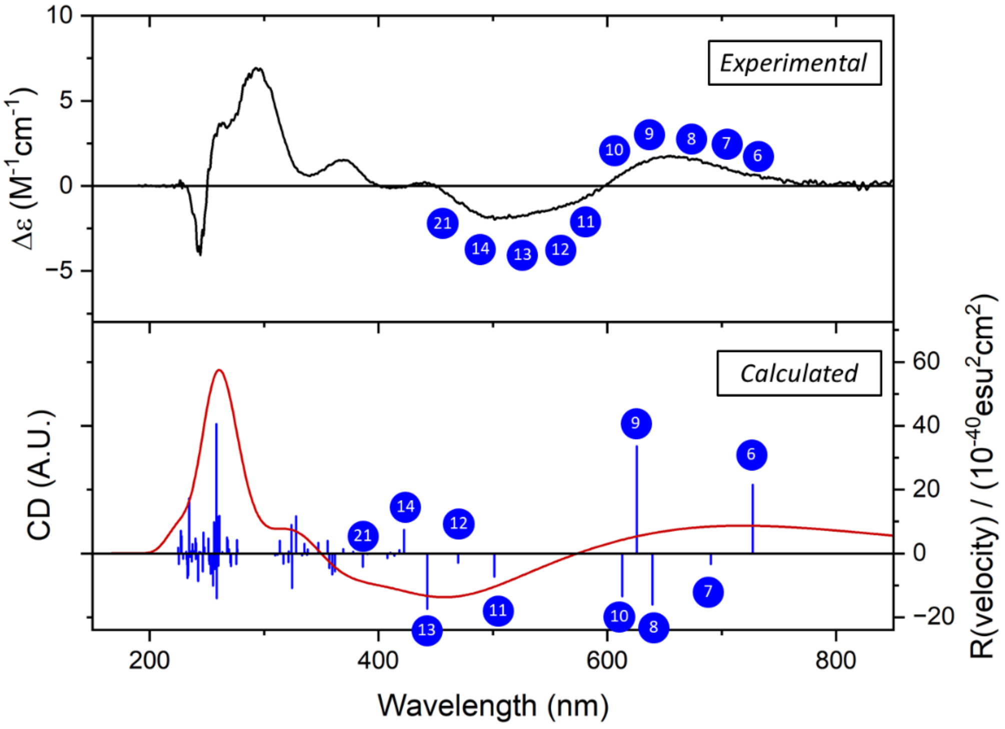

Figure S8. Experimental (top) and calculated (bottom) ECD spectra for **RuNCS** in dichloromethane. The transitions involving the bimetallic unit used for the assignment of the absorption spectra are labelled with their respective number.

Table S2. Tentative assignment of the most relevant absorption bands (nm) of (*S*)-**RuCl** and (*S*)-**RuNCS**.

Transition/complex	(*S*)-RuCl	(*S*)-RuNCS
**π(L) ⟶ π*(L)**	~246 sh	~246 sh
**π(L) ⟶ π*(L)**	277	277
**π(N/O/aryl) ⟶ σ*(Ru$_2$/axial)**	~375 sh	~399 sh
**π(axial) ⟶ π*(Ru$_2$)**<br>**π(aryl) ⟶ π*(Ru$_2$)**	~432 sh<br>471	498
**σ(Ru$_2$/axial) ⟶ δ*(Ru$_2$)**<br>**δ(Ru$_2$) ⟶ δ*(Ru$_2$)**<br>**π(aryl) ⟶ δ*(Ru$_2$)**	552	~551 sh
**π(Ru$_2$) ⟶ δ*(Ru$_2$)**<br>**σ(Ru$_2$) ⟶ π*(Ru$_2$)**	~633 sh	~640 sh

Single-crystal X-ray diffraction

Table S3. Crystallographic data of (*S*)-**RuCl**·0.5THF.

Empirical formula	$C_{36}H_{48}ClN_4O_{10.5}Ru_2S_2$
Formula weight	1006.49
Temperature/K	296.15
Crystal system	orthorhombic
Space group	$P2_12_12_1$
a/Å	13.3929(4)
b/Å	13.6437(5)
c/Å	24.6479(9)
α/°	90
β/°	90
γ/°	90
Volume/Å^3	4503.9(3)
Z	4
ρ_{calc}/g cm^{-3}	1.484
μ/mm^{-1}	0.878
F(000)	2052.0
Crystal size/mm^3	0.14 × 0.13 × 0.05
Radiation	MoKα (λ = 0.71073)
2Θ range for data collection/°	5.818 to 50.696
Index ranges	-16 ≤ h ≤ 15, -16 ≤ k ≤ 16, -29 ≤ l ≤ 29
Reflections collected	54642
Independent reflections	8131 [R_{int} = 0.0216, R_{sigma} = 0.0136]
Data/restraints/parameters	8131/12/529
Goodness-of-fit on F^2	1.086
Final R indexes [I>=2σ (I)]	R_1 = 0.0181, wR_2 = 0.0483
Final R indexes [all data]	R_1 = 0.0188, wR_2 = 0.0488
Largest diff. peak/hole / e Å^{-3}	0.26/-0.26
Flack parameter	-0.032(5)

Table S4. Crystallographic data of (*R*)-**RuCl**·0.5THF.

Empirical formula	$C_{36}H_{48}ClN_4O_{10.5}Ru_2S_2$
Formula weight	1006.49
Temperature/K	296.15
Crystal system	orthorhombic
Space group	$P2_12_12_1$
a/Å	13.402(3)
b/Å	13.646(4)
c/Å	24.592(5)
α/°	90
β/°	90
γ/°	90
Volume/Å^3	4497.6(18)
Z	4
ρ_{calc}/g cm^{-3}	1.486
μ/mm^{-1}	0.879
F(000)	2052.0
Crystal size/mm^3	0.16 × 0.15 × 0.05
Radiation	MoKα (λ = 0.71073)
2Θ range for data collection/°	4.26 to 50.7
Index ranges	-16 ≤ h ≤ 15, -16 ≤ k ≤ 16, -29 ≤ l ≤ 28
Reflections collected	36438
Independent reflections	8161 [R_{int} = 0.0306, R_{sigma} = 0.0251]
Data/restraints/parameters	8161/42/529
Goodness-of-fit on F^2	1.128
Final R indexes [I>=2σ (I)]	R_1 = 0.0268, wR_2 = 0.0684
Final R indexes [all data]	R_1 = 0.0285, wR_2 = 0.0690
Largest diff. peak/hole / e Å^{-3}	0.45/-0.26
Flack parameter	-0.011(10)

Table S5. Crystallographic data of (*S*)-**RuNCS**·0.5toluene·solvent (solvent = cyclohexane).

Empirical formula*	$C_{38.5}H_{48}N_5O_{10}Ru_2S_3$
Formula weight*	1039.14
Temperature/K	100.0
Crystal system	orthorhombic
Space group	$P2_12_12_1$
a/Å	13.4136(6)
b/Å	27.358(2)
c/Å	29.596(2)
α/°	90
β/°	90
γ/°	90
Volume/Å^3	10861.0(13)
Z	8
ρ_{calc}/g cm^{-3}	1.271
μ/mm^{-1}	5.989
F(000)	4240.0
Crystal size/mm^3	0.128 × 0.034 × 0.031
Radiation	CuKα (λ = 1.54178)
2Θ range for data collection/°	4.398 to 136.484
Index ranges	-13 ≤ h ≤ 16, -32 ≤ k ≤ 32, -35 ≤ l ≤ 35
Reflections collected	83334
Independent reflections	19650 [R_{int} = 0.2125, R_{sigma} = 0.1587]
Data/restraints/parameters	19650/0/1061
Goodness-of-fit on F^2	0.959
Final R indexes [I>=2σ (I)]	R_1 = 0.0680, wR_2 = 0.1427
Final R indexes [all data]	R_1 = 0.1031, wR_2 = 0.1583
Largest diff. peak/hole / e Å^{-3}	0.90/-1.18
Flack parameter	0.023(14)

* The 1.85 cyclohexane solvent molecules estimated by the solvent mask routine implemented in OLEX are not included in the empirical formula.

Table S6. Crystallographic data of (*R*)-**RuNCS**·0.5toluene·solvent (solvent = cyclohexane).

Empirical formula*	$C_{38.5}H_{48}N_5O_{10}Ru_2S_3$
Formula weight*	1039.14
Temperature/K	100.03(12)
Crystal system	orthorhombic
Space group	$P2_12_12_1$
a/Å	13.3749(2)
b/Å	27.3506(5)
c/Å	29.5618(6)
α/°	90
β/°	90
γ/°	90
Volume/Å^3	10814.0(3)
Z	8
ρ_{calc}/g cm^{-3}	1.277
μ/mm^{-1}	0.723
F(000)	4240.0
Crystal size/mm^3	0.3 × 0.07 × 0.06
Radiation	MoKα (λ = 0.71073)
2Θ range for data collection/°	4.058 to 50.742
Index ranges	$-16 \leq h \leq 16$, $-32 \leq k \leq 32$, $-35 \leq l \leq 35$
Reflections collected	100394
Independent reflections	19796 [R_{int} = 0.0597, R_{sigma} = 0.0410]
Data/restraints/parameters	19796/0/1055
Goodness-of-fit on F^2	1.035
Final R indexes [I>=2σ (I)]	R_1 = 0.0352, wR_2 = 0.0804
Final R indexes [all data]	R_1 = 0.0440, wR_2 = 0.0846
Largest diff. peak/hole / e Å^{-3}	1.25/-0.48
Flack parameter	-0.004(11)

* The 1.57 cyclohexane solvent molecules estimated by the solvent mask routine implemented in OLEX are not included in the empirical formula.

Table S7. Selected bond distances (Å) and angles (deg) for (*S*)-**RuCl**·0.5THF.

Ru1-Ru2	2.2904(3)	Ru2-N4	2.015(3)	Cl1-Ru1-Ru2	170.51(2)
Ru1-Cl1	2.4492(8)	Ru1-O7	2.058(2)	N1-Ru1-O7	176.8(1)
Ru1-N1	2.075(3)	Ru2-O8	2.034(2)	N2-Ru2-O8	176.7(1)
Ru2-N2	2.035(3)	Ru1-O9	2.081(2)	N3-Ru1-O9	178.1(1)
Ru1-N3	2.060(3)	Ru2-O10	2.037(2)	N4-Ru2-O10	174.7(1)
N1-Ru1-Ru2-N2		3.7(1)	N3-Ru1-Ru2-N4		5.7(1)
O7-Ru1-Ru2-O8		2.42(9)	O9-Ru1-Ru2-O10		0.93(9)

Table S8. Selected bond distances (Å) and angles (deg) for (*R*)-**RuCl**·0.5THF.

Ru1-Ru2	2.2906(6)	Ru2-N4	2.039(4)	Cl1-Ru1-Ru2	170.50(4)
Ru1-Cl1	2.450(1)	Ru1-O7	2.080(4)	N1-Ru1-O7	178.1(2)
Ru1-N1	2.062(4)	Ru2-O8	2.038(3)	N2-Ru2-O8	174.8(2)
Ru2-N2	2.023(4)	Ru1-O9	2.062(3)	N3-Ru1-O9	176.9(2)
Ru1-N3	2.075(4)	Ru2-O10	2.034(4)	N4-Ru2-O10	176.7(2)
N1-Ru1-Ru2-N2		5.6(2)	N3-Ru1-Ru2-N4		3.9(2)
O7-Ru1-Ru2-O8		0.9(1)	O8-Ru1-Ru2-O10		2.3(1)

Table S9. Selected bond distances (Å) and angles (deg) for (*S*)-**RuNCS**·0.5toluene·solvent.

Ru1-Ru2	2.289(1)	Ru2-N4	2.01(1)	N5-Ru1-Ru2	171.9(3)
Ru1-N5	2.15(1)	Ru1-O7	2.07(1)	N1-Ru1-O7	179.0(4)
Ru1-N1	2.10(1)	Ru2-O8	2.05(1)	N2-Ru2-O8	173.8(4)
Ru2-N2	2.03(1)	Ru1-O9	2.06(1)	N3-Ru1-O9	177.1(5)
Ru1-N3	2.05(1)	Ru2-O10	2.048(8)	N4-Ru2-O10	175.7(5)
Ru3-Ru4	2.285(1)	Ru4-N9	2.02(1)	N10-Ru3-Ru4	172.9(3)
Ru3-N10	2.14(1)	Ru3-O17	2.066(9)	N6-Ru3-O17	179.0(5)
Ru3-N6	2.04(1)	Ru4-O18	2.045(9)	N7-Ru4-O18	177.3(4)
Ru4-N7	2.06(1)	Ru3-O19	2.07(1)	N8-Ru3-O19	175.4(4)
Ru3-N8	2.07(1)	Ru4-O20	2.05(1)	N9-Ru4-O20	176.8(5)
				Ru1-N5-C35	148(1)
				Ru3-N10-C71	150(1)
N1-Ru1-Ru2-N2		6.7(4)	N3-Ru1-Ru2-N4		0.8(4)
O7-Ru1-Ru2-O8		1.0(4)	O9-Ru1-Ru2-O10		1.3(4)
N6-Ru3-Ru4-N7		4.7(4)	N8-Ru3-Ru4-N9		12.6(5)
O17-Ru3-Ru4-O18		6.6(4)	O19-Ru3-Ru4-O20		5.2(4)

Table S10. Selected bond distances (Å) and angles (deg) for (*R*)-**RuNCS**·0.5toluene·solvent.

Ru1-Ru2	2.2861(6)	Ru2-N4	2.012(4)	N5-Ru1-Ru2	172.2(1)
Ru1-N5	2.15(1)	Ru1-O7	2.045(4)	N1-Ru1-O7	179.6(2)
Ru1-N1	2.079(1)	Ru2-O8	2.031(4)	N2-Ru2-O8	173.8(2)
Ru2-N2	2.033(5)	Ru1-O9	2.087(4)	N3-Ru1-O9	177.7(2)
Ru1-N3	2.050(5)	Ru2-O10	2.052(4)	N4-Ru2-O10	176.0(2)
Ru3-Ru4	2.2869(7)	Ru4-N9	2.028(5)	N10-Ru3-Ru4	172.8(1)
Ru3-N10	2.153(5)	Ru3-O17	2.069(4)	N6-Ru3-O17	178.1(2)
Ru3-N6	2.063(5)	Ru4-O18	2.043(4)	N8-Ru4-O19	176.5(2)
Ru4-N7	2.044(5)	Ru3-O19	2.059(4)	O18-Ru4-N7	176.9(1)
Ru3-N8	2.077(5)	Ru4-O20	2.024(4)	O20-Ru4-N9	176.4(2)
				Ru1-N5-C36	149.2(5)
				Ru3-N10-C71	150.5(5)
N1-Ru1-Ru2-N2		6.7(2)	N3-Ru1-Ru2-N4		0.6(2)
O7-Ru1-Ru2-O8		0.7(2)	O9-Ru1-Ru2-O10		1.4(2)
N6-Ru3-Ru4-N7		4.8(2)	N8-Ru3-Ru4-N9		11.7(2)
O17-Ru3-Ru4-O18		6.2(2)	O19-Ru3-Ru4-O20		5.0(2)

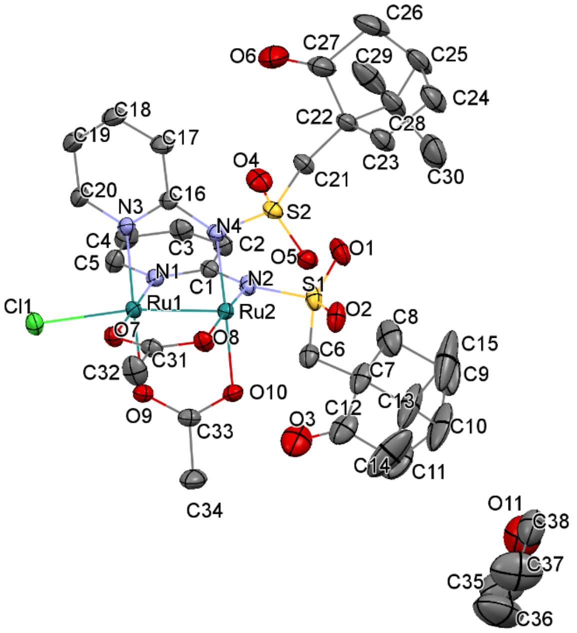

Figure S9. Asymmetric unit of (*S*)-**RuCl**·0.5THF. Anisotropic displacement ellipsoids are drawn at the 30% probability level. Hydrogen atoms have been omitted for clarity.

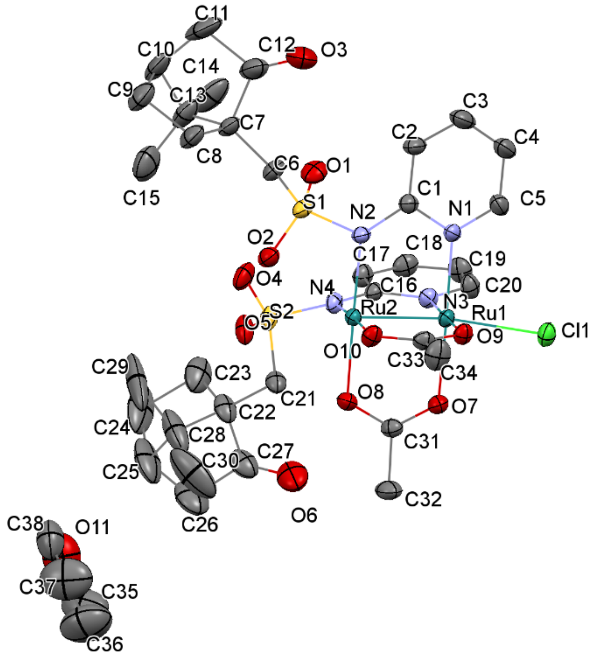

Figure S10. Asymmetric unit of (*R*)-**RuCl**·0.5THF. Anisotropic displacement ellipsoids are drawn at the 30% probability level. Hydrogen atoms have been omitted for clarity.

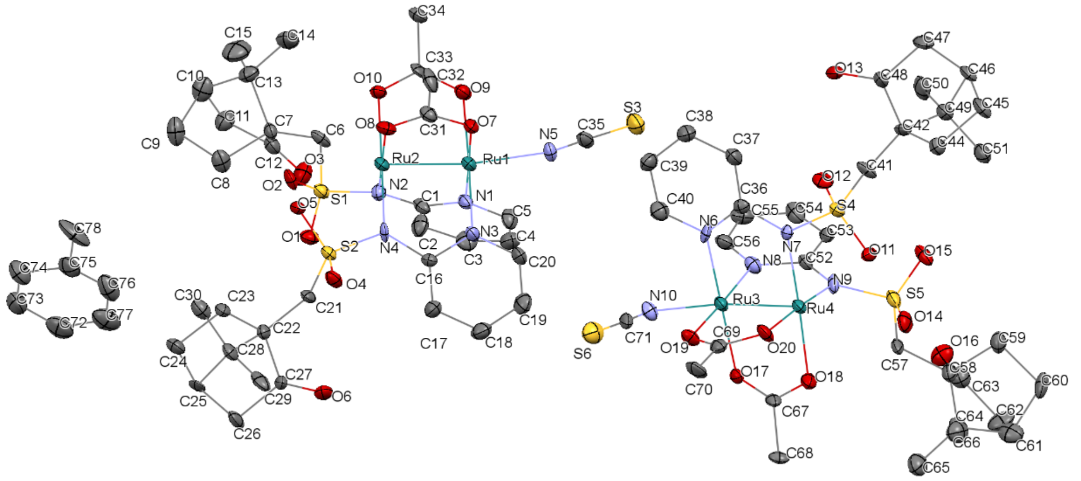

Figure S11. Asymmetric unit of (*S*)-**RuNCS**·0.5toluene·solvent (solvent = cyclohexane). Anisotropic displacement ellipsoids are drawn at the 30% probability level. Hydrogen atoms have been omitted for clarity.

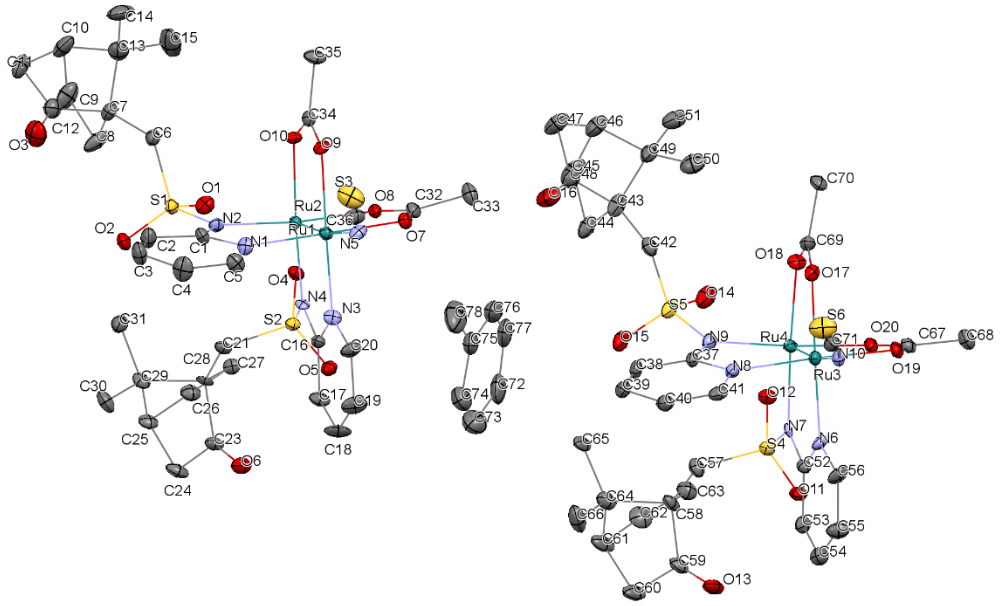

Figure S12. Asymmetric unit of (*R*)-**RuNCS**·0.5toluene·solvent (solvent = cyclohexane). Anisotropic displacement ellipsoids are drawn at the 30% probability level. Hydrogen atoms have been omitted for clarity.

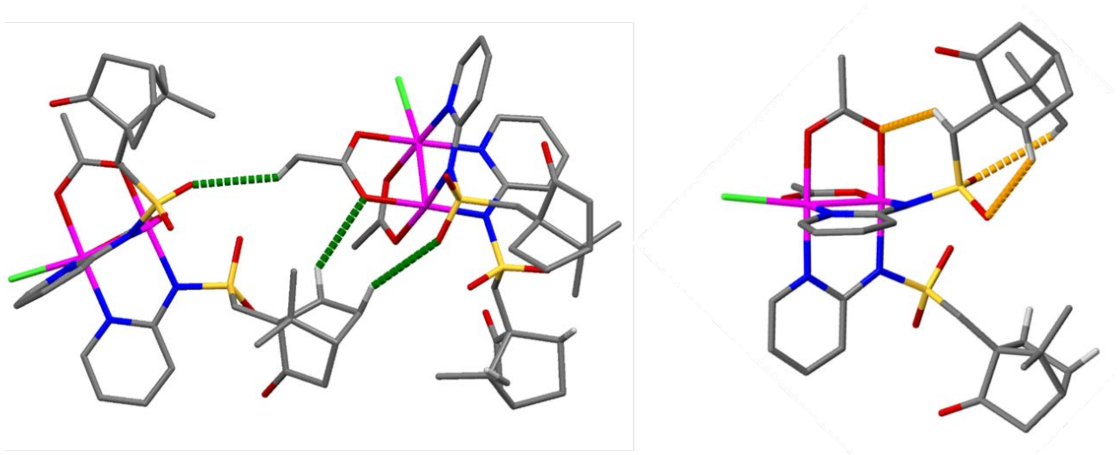

Figure S13. Inter- (dark green) (left) and intramolecular (orange) (right) C-H···O interactions found in the structure of (S)-**RuCl**·0.5THF. H atoms not involved in the interactions are omitted for the sake of clarity.

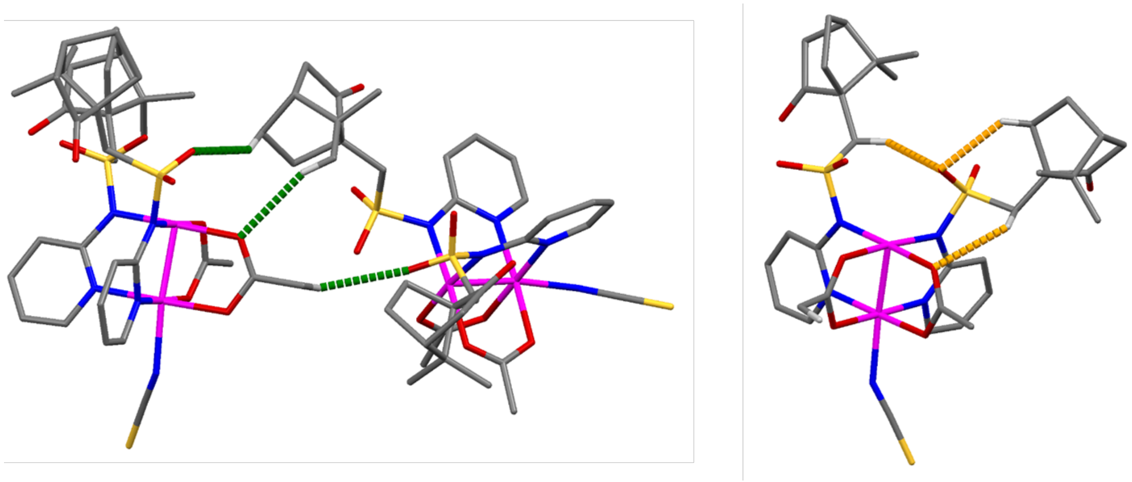

Figure S14. Inter- (dark green) (left) and intramolecular (orange) (right) C-H···O interactions found in the structure of (*S*)-**RuNCS**·0.5toluene·solvent. H atoms not involved in the interactions are omitted for the sake of clarity.

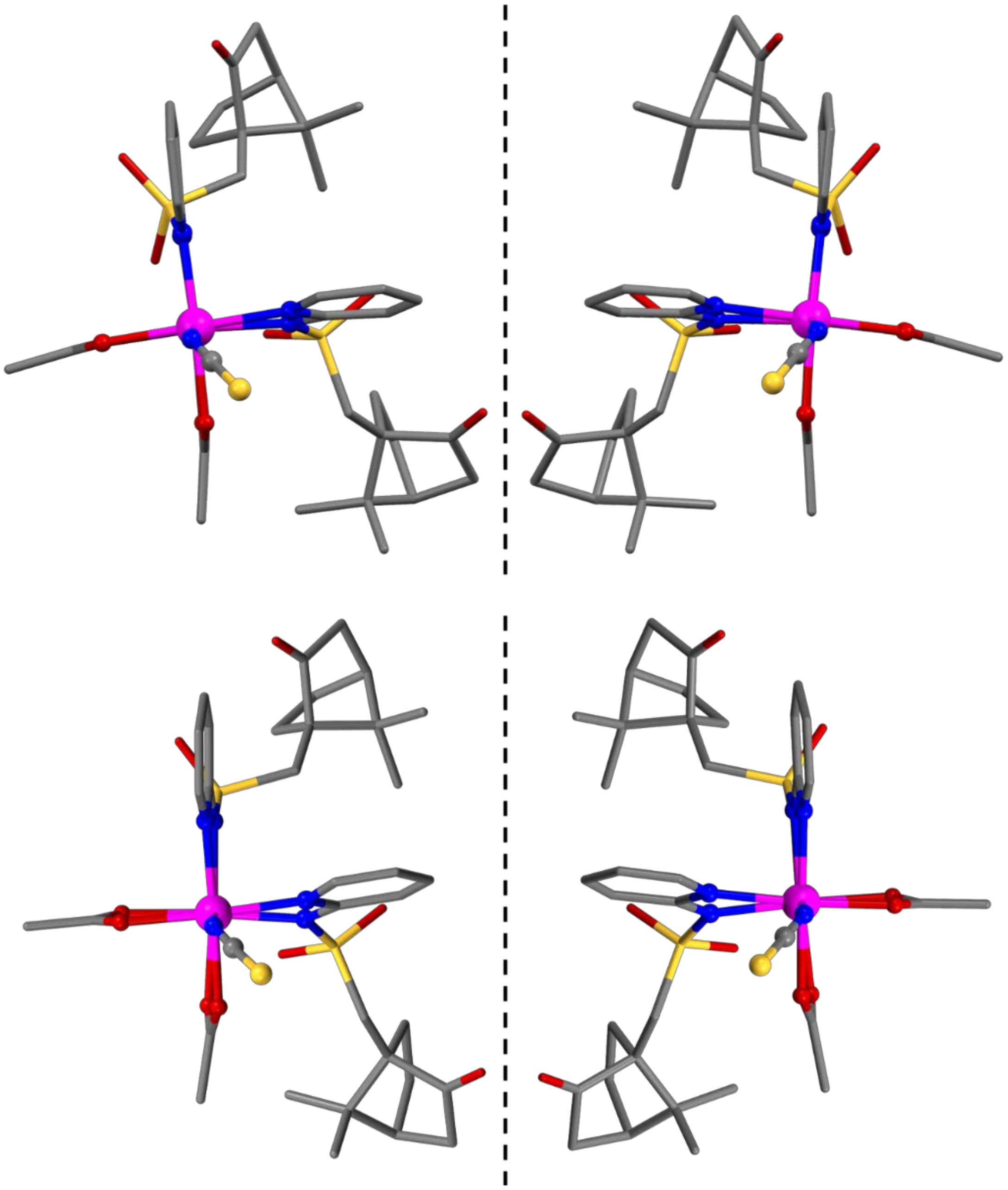

(*R*)-**RuNCS** (*S*)-**RuNCS**

Figure S15. View of the crystal structure along the Ru-Ru axis for the two molecules present in the unit cell for (*R*)-**RuNCS** (left) and (*S*)-**RuNCS** (right), showing the overall conformation of the paddlewheel motif that is close to eclipsed.

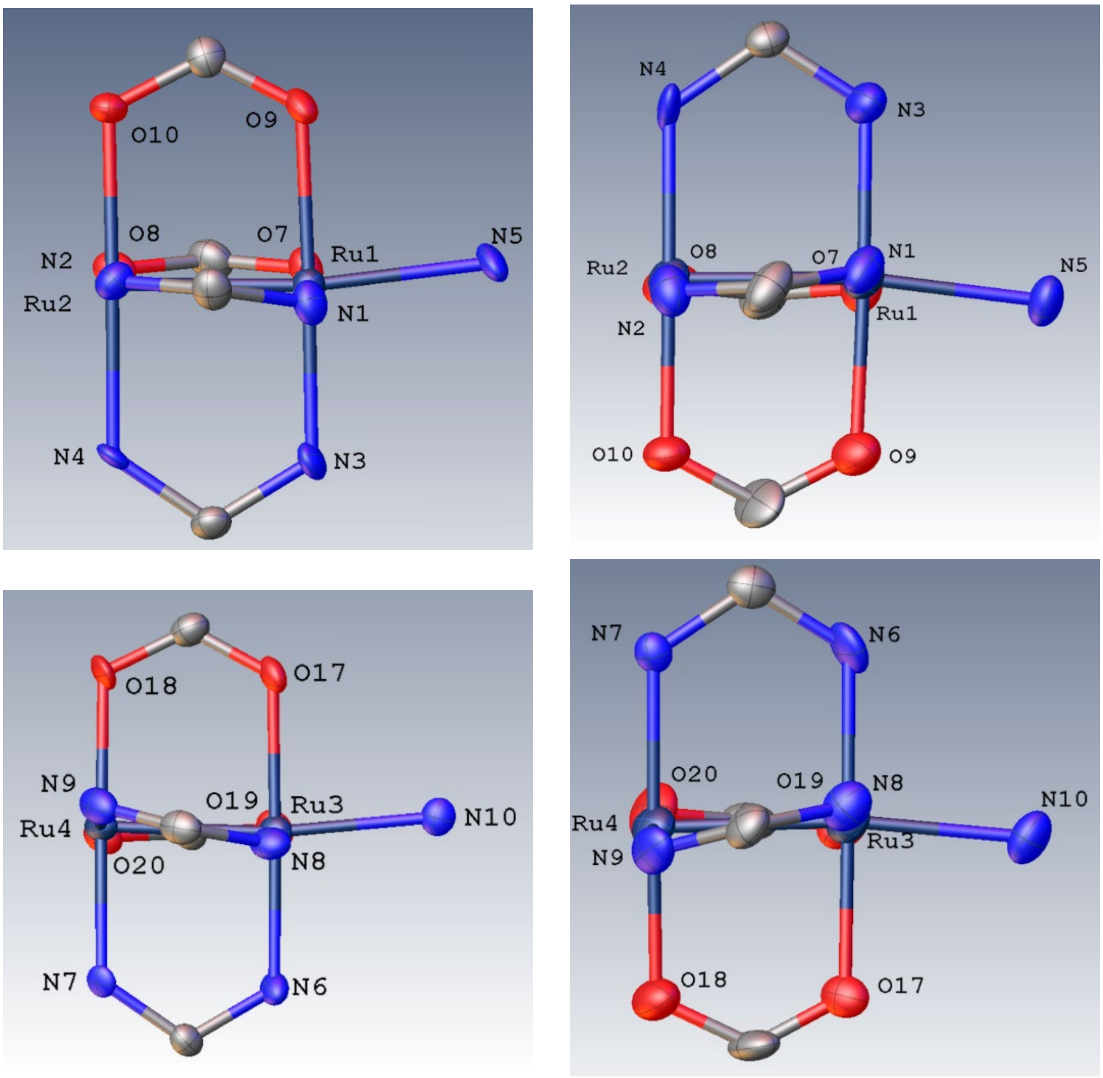

Figure S16. View of the diruthenium paddlewheel motif perpendicular to the Ru-Ru axis for the two molecules present in the unit cell for (*R*)-**RuNCS** (left) and (*S*)-**RuNCS** (right).

Table S11. Angles between the Ru-Ru axis and the mean planes best fit for each E-C-E (E = N, O) paddle/ligand were calculated with the crystallographic Olex2 software, following the standard IUPAC nomenclature convention (see Jensen, K. A. *Inorg. Chem.* **1970**, *9*, 1).

metal-metal	ligand plane definition	angle
(*R*)-RuNCS or (Δ)-RuNCS		
Ru1-Ru2	N1 C1 N2	+6.07(18)
Ru1-Ru2	N3 C16 N4	-0.47(16)
Ru1-Ru2	O7 C32 O8	+0.63(15)
Ru1-Ru2	O9 C34 O10	+1.33(14)
Ru3-Ru4	N6 C52 N7	+4.37(16)
Ru3-Ru4	N8 C37 N9	+10.29(19)
Ru3-Ru4	O17 C69 O18	+5.76(14)
Ru3-Ru4	O19 C67 O20	+4.50(15)
(*S*)-RuNCS or (Λ)-RuNCS		
Ru1-Ru2	N1 C1 N2	-6.2(4)
Ru1-Ru2	N3 C16 N4	+0.6(4)
Ru1-Ru2	O7 C31 O8	-0.9(4)
Ru1-Ru2	O9 C33 O10	-1.2(4)
Ru3-Ru4	N6 C36 N7	-4.2(4)
Ru3-Ru4	N8 C52 N9	-11.0(4)
Ru3-Ru4	O17 C67 O19	-6.1(4)
Ru3-Ru4	O19 C69 O20	-4.7(4)

Magnetic measurements

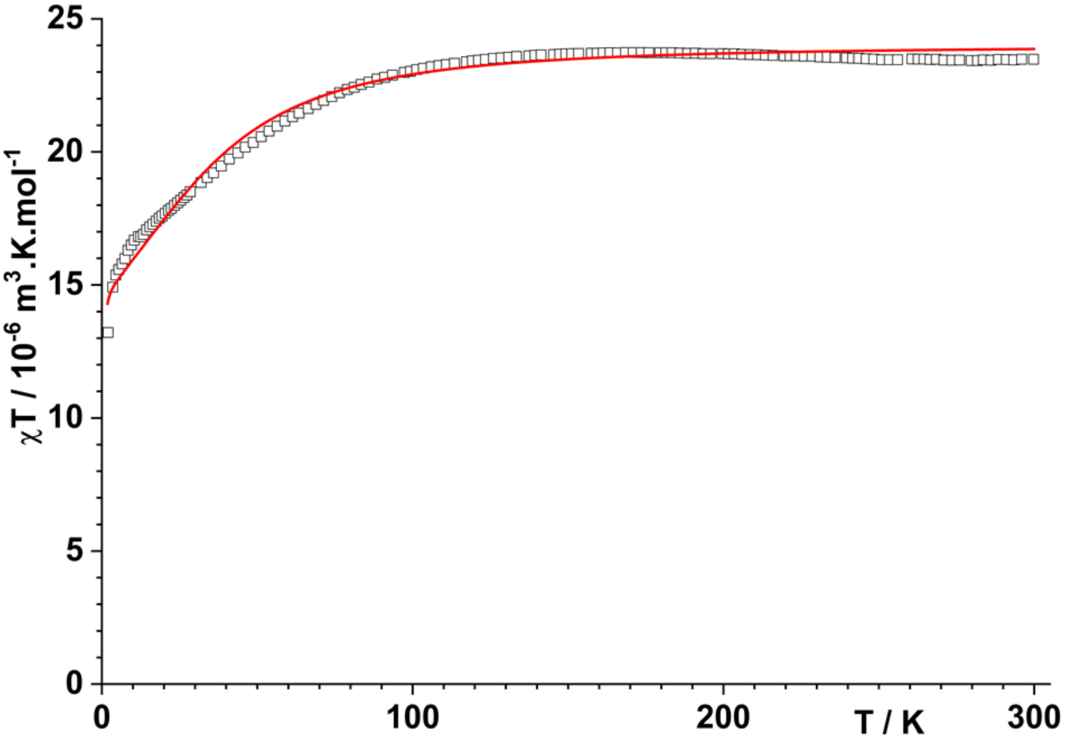

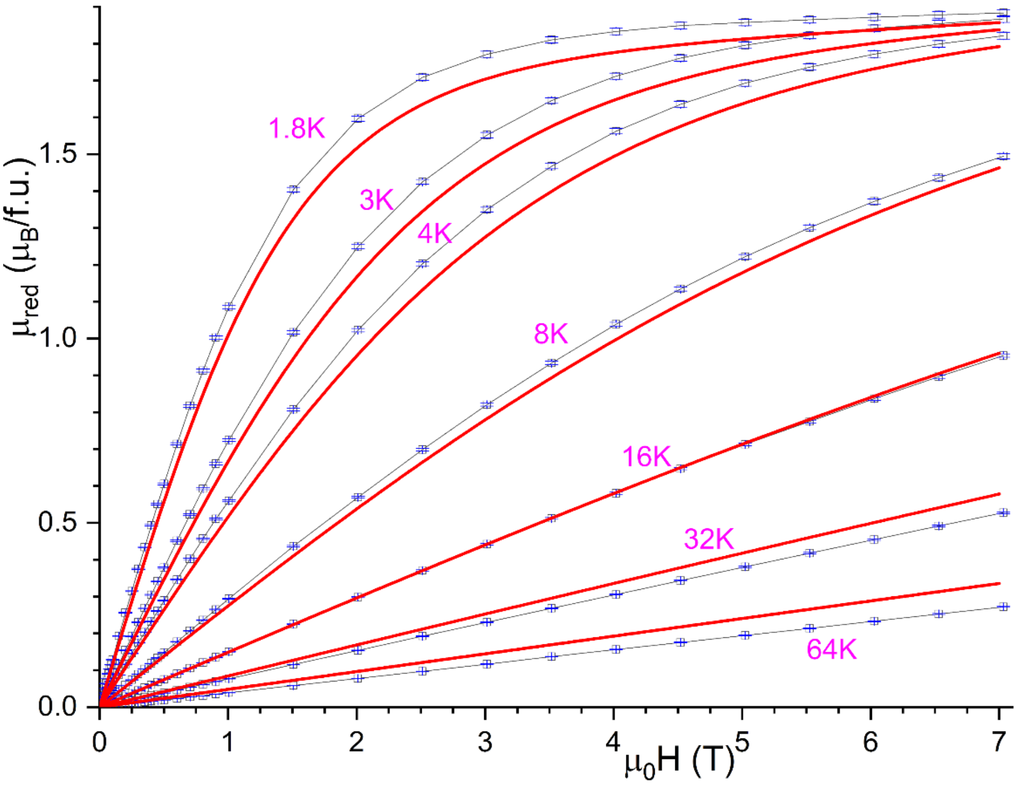

Figure S17. (top) Temperature dependence of the product of molar susceptibility with temperature $\chi_M T$ (squares) for (*S*)-**RuCl**; (bottom) Field dependence of isothermal magnetization between 1.8 and 64 K (squares). Red solid lines are the best simultaneous fit to the data using the PHI software with parameters reported in Table S12.

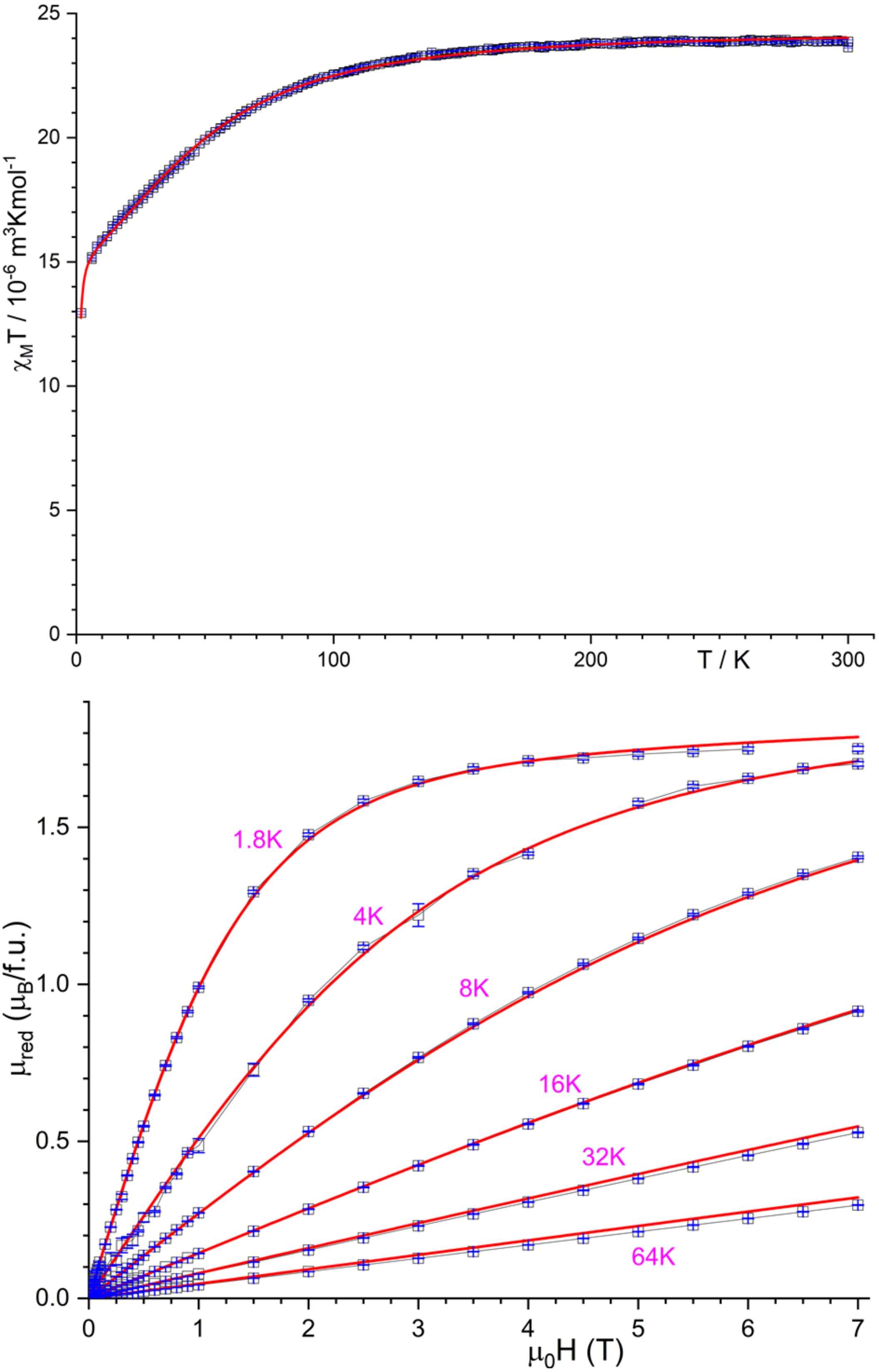

Figure S18. (top) Temperature dependence of the product of molar susceptibility with temperature $\chi_M T$ (squares) for (*S*)-**RuNCS**; (bottom) Field dependence of isothermal magnetization between 1.8 and 64 K (squares). Red solid lines are the best simultaneous fit to the data using the PHI software with parameters reported in Table S12.

Table S12. PHI best parameters obtained from the simultaneous fitting of the magnetic data of (*S*)-**RuCl** and (*S*)-**RuNCS**. Parameters were incorporated gradually monitoring the absence of excessive correlation between them. *D*, *E* and *zJ* follow equations (2.2.15), (2.2.16) and (2.2.53) described in the PHI users manual v3. No significant contribution of temperature-independent paramagnetism was found.

**Compound**	(*S*)-**RuCl**	(*S*)-**RuNCS**
$\chi_M T$ at 300 K (10^{-6} m^3·K·mol^{-1})	23.48	23.63
g	2.0181(8)	2.0288(2)
D (K)	62.9(5)	78.7(2)
E (K)	--	-17.9(2)
zJ (K)	0.008(4)	0.037(2)
***Res*(*)**	0.0169	2.26×10^{-4}

Cyclic voltammetry

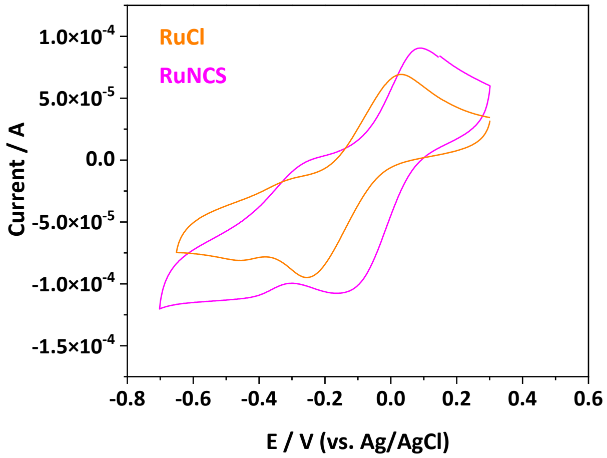

Figure S19. CV data of TBAP 0.05 M dichloromethane solutions of (*S*)-**RuCl** (orange) and (*S*)-**RuNCS** (pink).

Table S13. Electrochemical data (V, vs. Ag/AgCl) from CV of (*S*)-**RuCl** and (*S*)-**RuNCS**.

**Process/ Compound**		**RuCl**	**RuNCS**
$Ru_2^{5+/4+}$	**$E_{1/2}$**	-0.11	-0.03
	**ΔE**	0.29	0.23

Complex stability

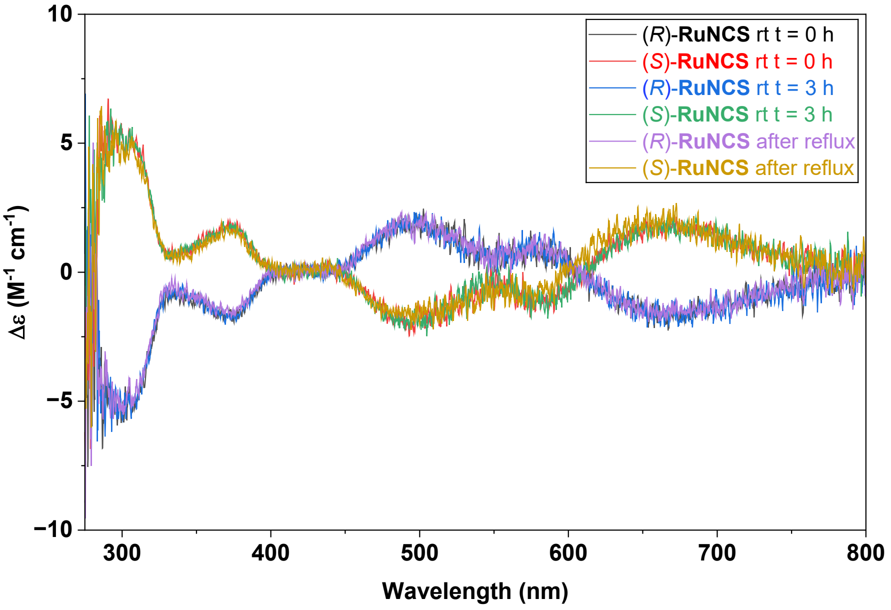

Figure S20. CD spectra of (*R*)- and (*S*)-**RuNCS** toluene solutions in different conditions.

Surface characterization

Atomic Force Microscopy

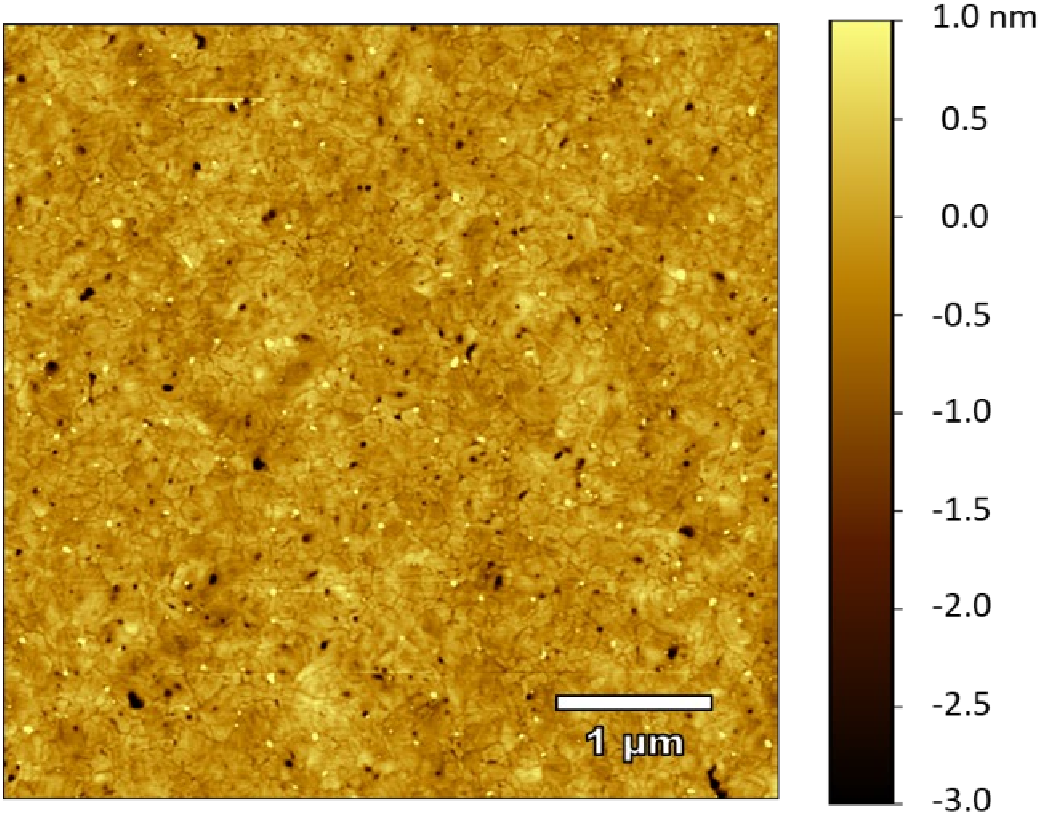

Figure S21. Tapping-mode AFM image for a blank Au^{TS} reproduced from ref. 5.[5]

Time-of-flight secondary ion mass spectrometry (ToF-SIMS)

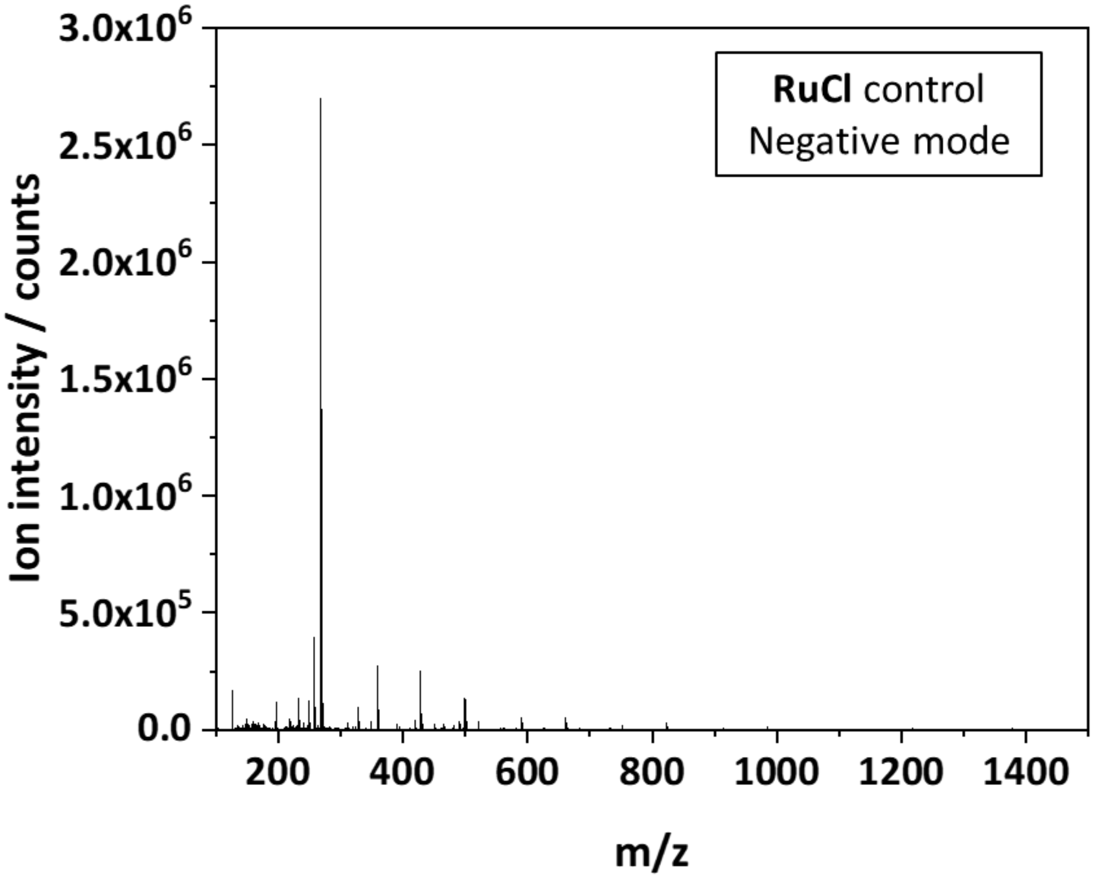

Figure S22. ToF-SIMS spectrum of ions derived from Au-(*S*)-**RuCl** in negative mode.

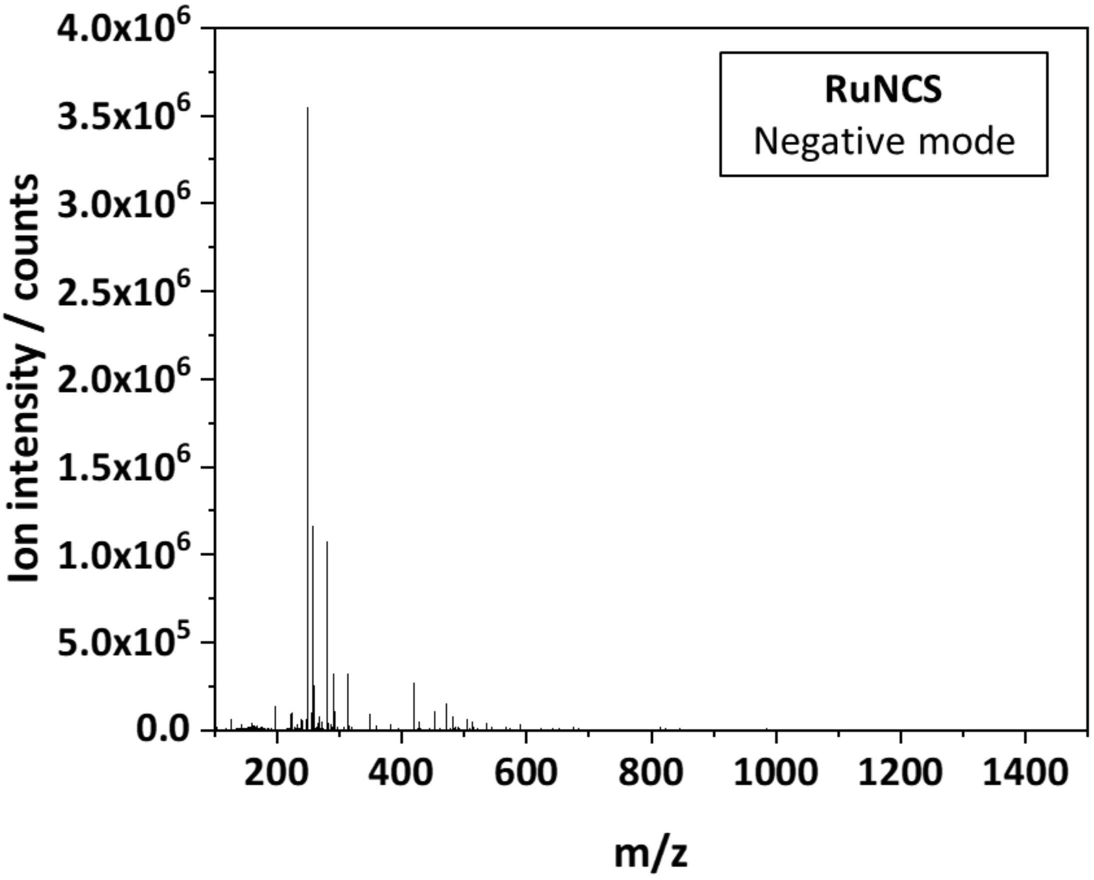

Figure S23. ToF-SIMS spectrum of ions derived from Au-(*S*)-**RuNCS** in negative mode.

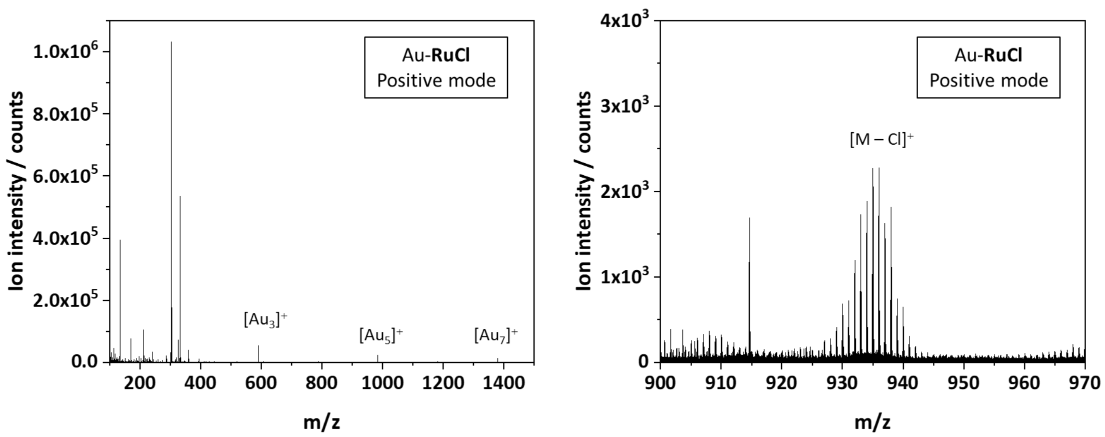

Figure S24. ToF-SIMS spectrum of ions derived from a Au substrate incubated in a (*S*)-**RuCl** solution in positive mode (left) and an enlargement of the spectrum (right).

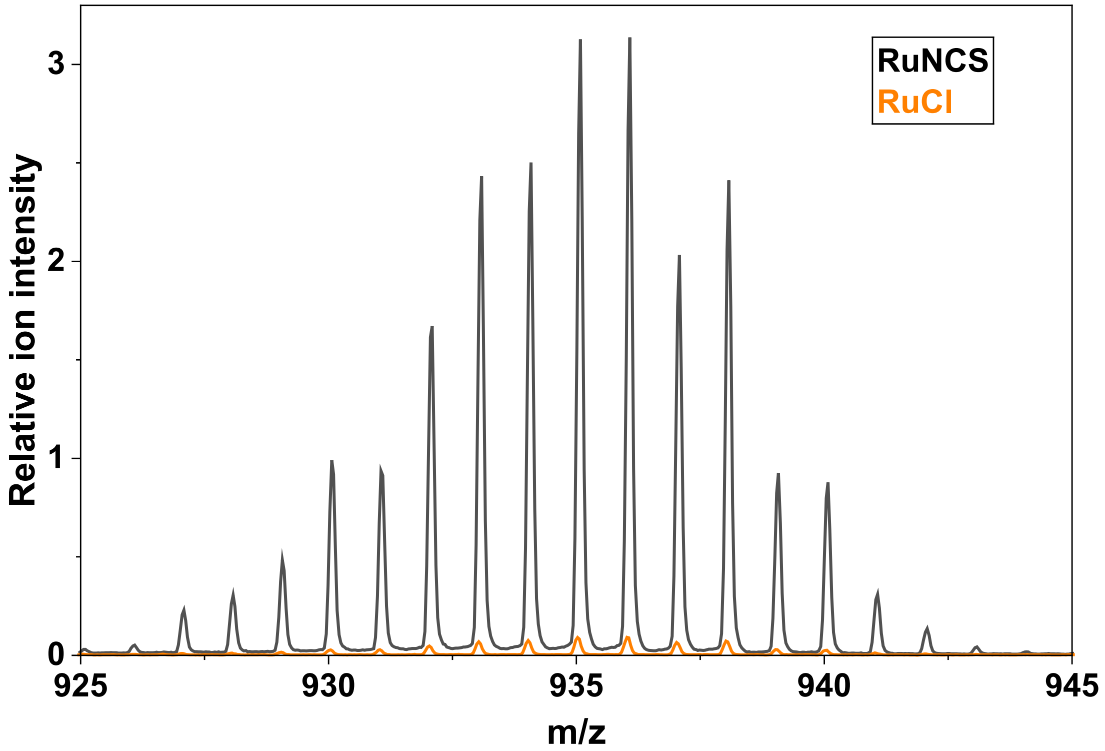

Figure S25. Comparison of the ion intensity of the $[M - X]^+$ fragment normalized to the intensity of the $[Au_5]^+$ fragment registered for Au incubated in a solution of (*S*)-**RuCl** (orange) and Au-(*S*)-**RuNCS** (black).

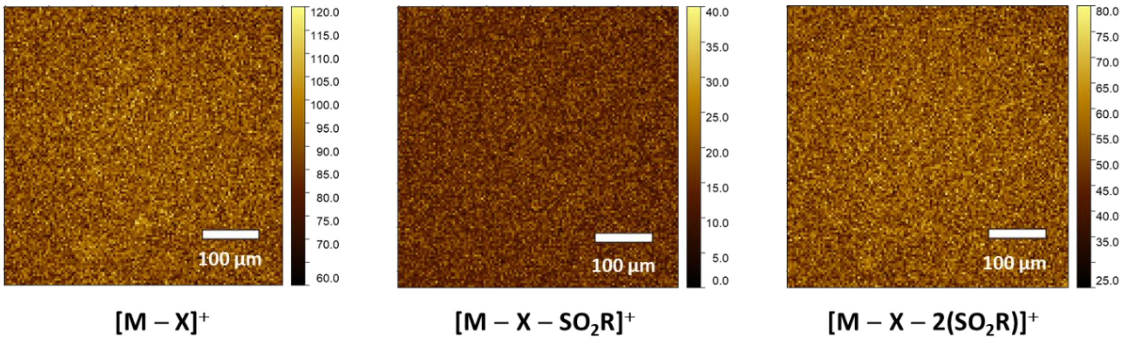

Figure S26. Two-dimensional mapping of positive $[M - X]^+$, $[M - X - SO_2R]^+$ and $[M - X - 2(SO_2R)]^+$ ions derived from Au-(*S*)-**RuNCS**. The bar on the right expresses counts. X refers to the NCS axial ligand and R to the camphor moiety of the pycsa ligand.

X-ray photoelectron spectroscopy (XPS)

Table S14. Semi-quantitative elemental analysis extracted from XPS characterization on (*S*)-**RuNCS** bulk sample as well as on Au-**RuNCS** SAM. The theoretical values correspond to the atomic percentage in number of atoms normalized to the atoms studied (2 Ru, 5 N and 3 S).

Sample	N1*s*	Ru3*d*	S2*p*	Ru/N	Ru/S	N/S
**Theoretical (%)**	50	20	30	0.4	0.7	1.7
**Bulk (%)**	48.8 ± 2.4	23 ± 1.2	28.2 ± 1.4	0.5	0.8	1.7
**SAM (%)**	50.5 ± 2.5	20.5 ± 1.0	29.0 ± 1.5	0.4	0.6	1.7

EGaIn and magnetic-conductive atomic force microscopy (mc-AFM) measurements

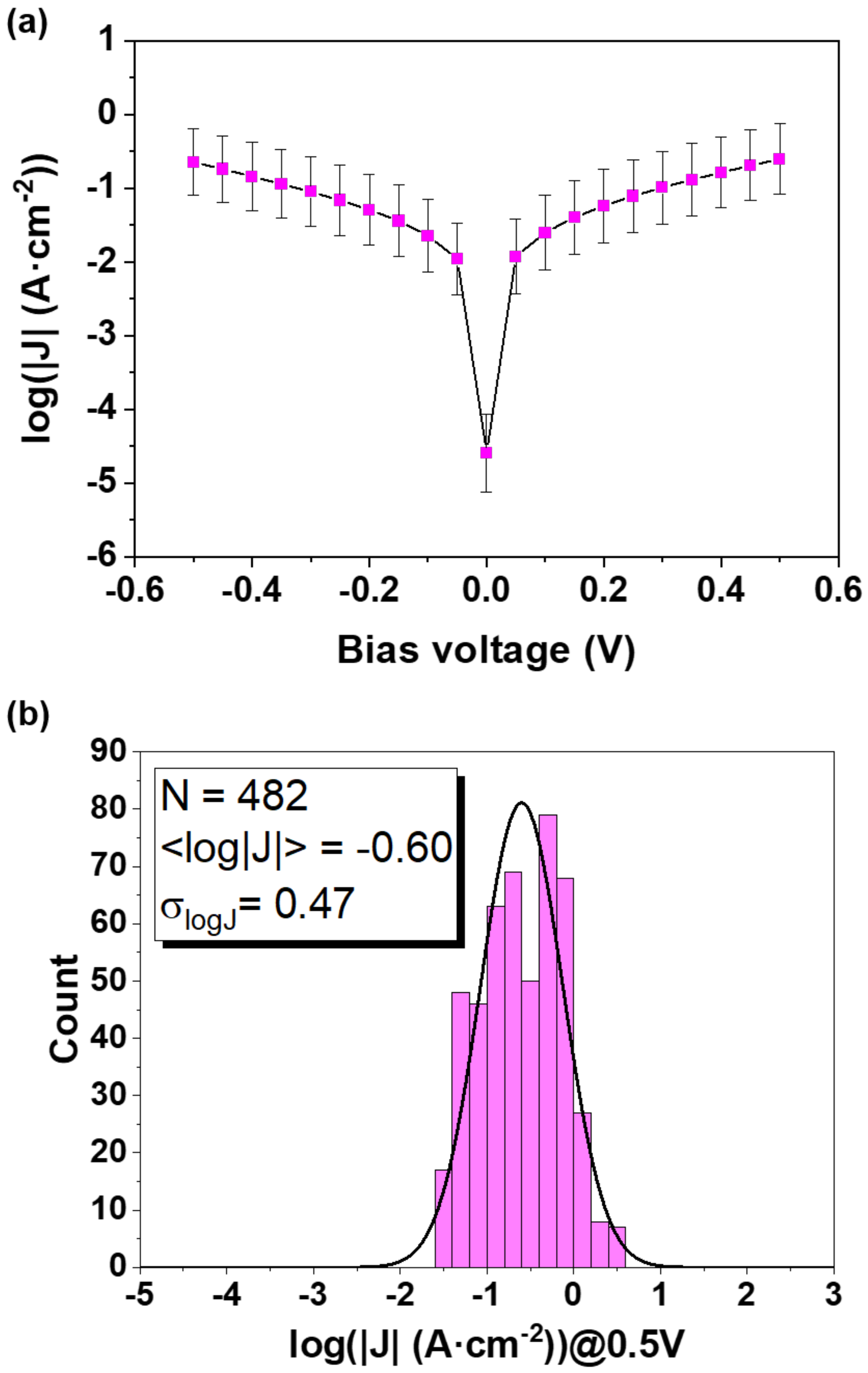

Figure S27. (a) Plot of <log(|*J*|)> versus applied bias voltage (V) for Au^{TS}-**RuNCS**//Ga_2O_3/EGaIn junctions (left). The error bars correspond to the standard deviation. (b) Histogram showing the distribution of log|*J*| values at *V* = +0.50 V for Au^{TS}-**RuNCS**//Ga_2O_3/EGaIn junctions (right).

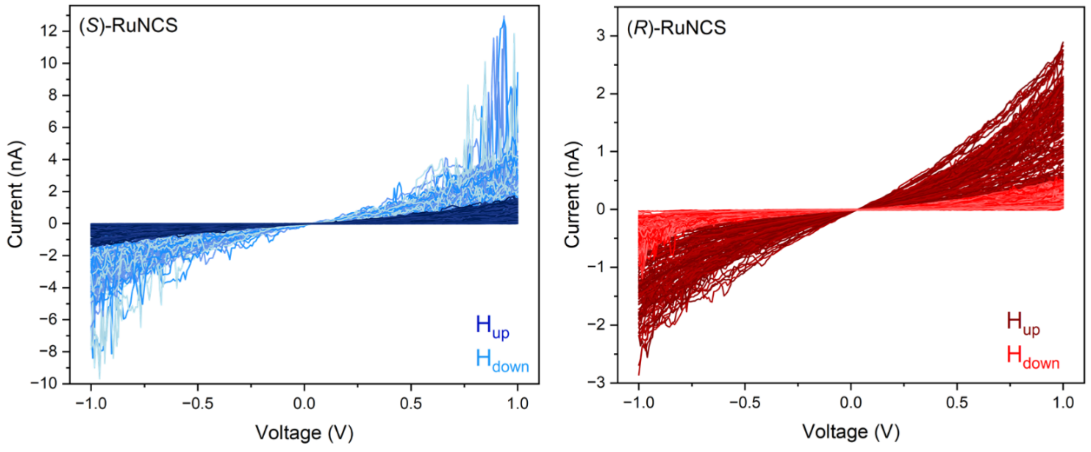

Figure S28. Complete dataset of mc-AFM measurements acquired on (*S*)-**RuNCS** (left) and on (*R*)-**RuNCS** (right) assembled on magnetic AuNi^{TS} electrode.

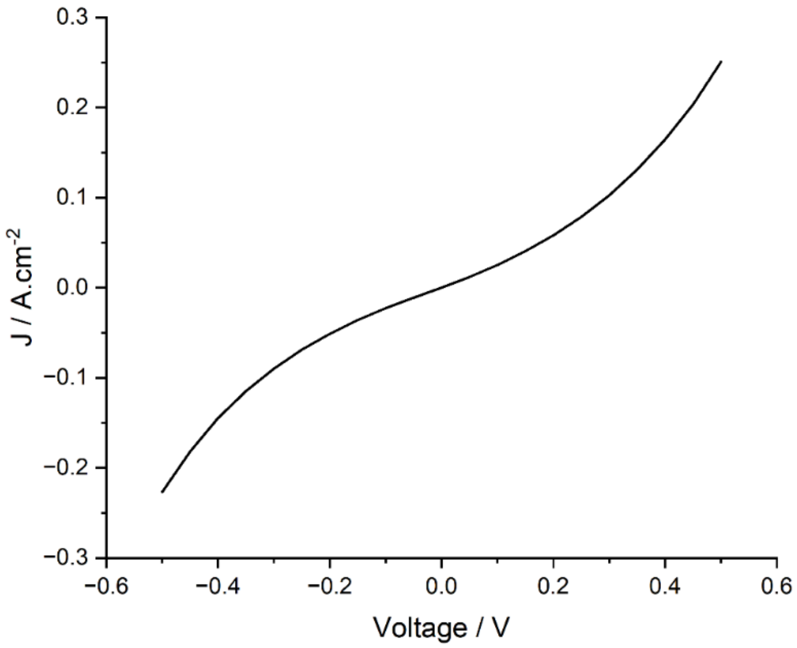

Figure S29. Average *J*/*V* curve measured by EGaIn large area junctions for Au^{TS}-(S)-**RuNCS**.

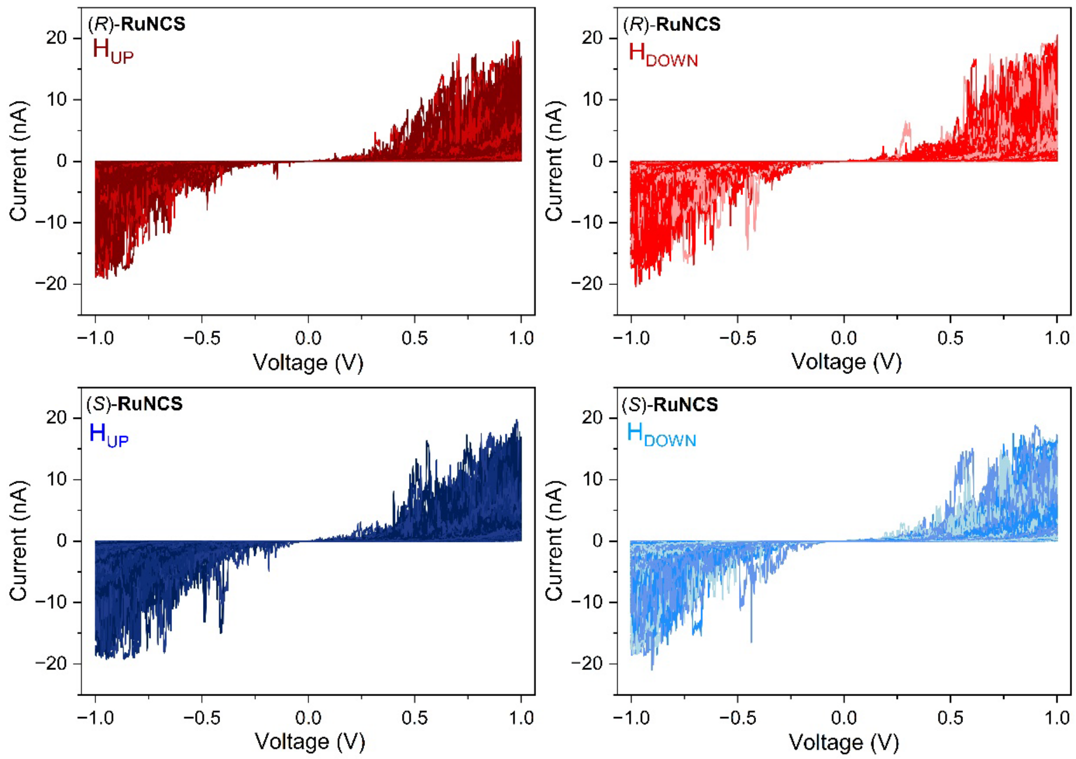

Figure S30. Complete dataset of mc-AFM measurements acquired on (*S*)-**RuNCS** (bottom) and on (*R*)-**RuNCS** (top) assembled on a diamagnetic Au^{TS} electrode.

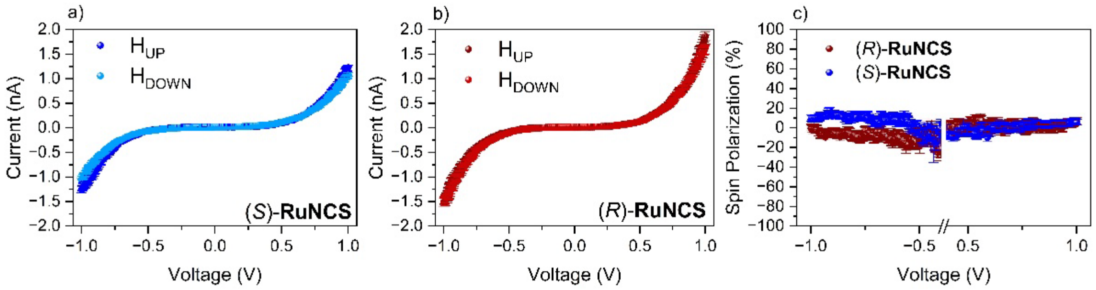

Figure S31. Averaged room temperature *I*/*V* curves acquired on (a) Au^{TS} (*S*)-**RuNCS** and on (b) Au^{TS} (*R*)-**RuNCS** applying in remanence either a positive or negative magnetic field of 0.5 T; (c) Spin polarization percentage extracted from the corresponding *I*/*V* curves for SAMs of both enantiomers at room temperature. The error bars correspond to the standard error $\sigma/\sqrt{n}$ where σ is the standard deviation and n is the number of measurements.

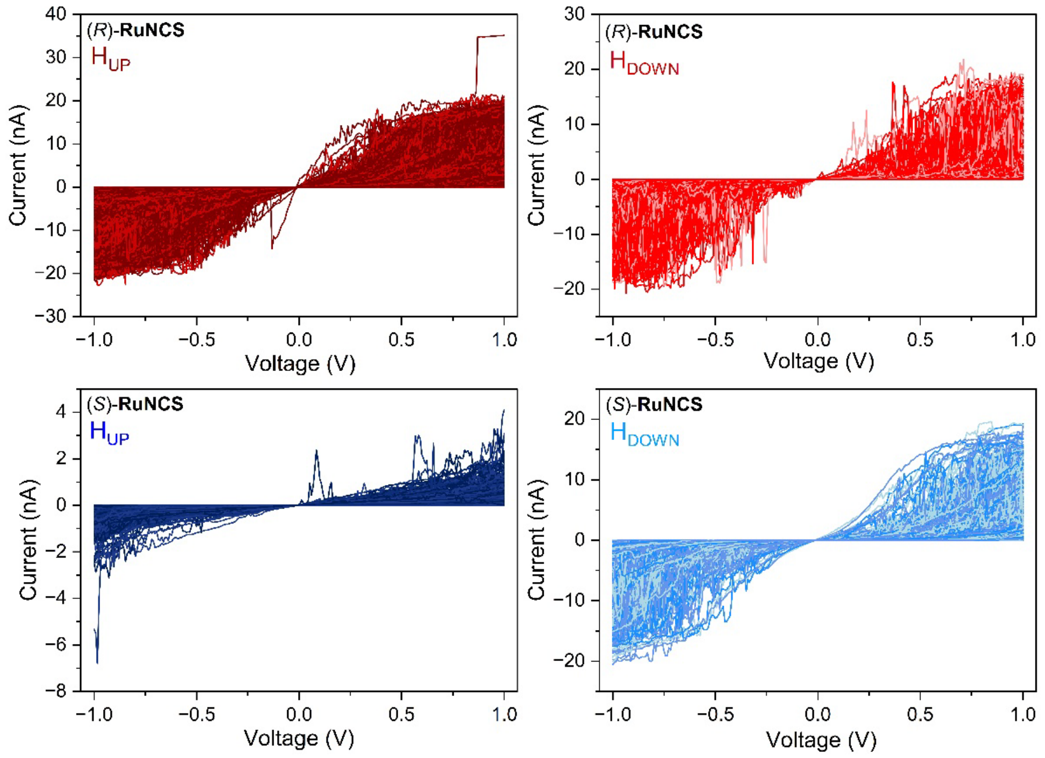

Figure S32. Complete dataset of mc-AFM measurements acquired on (*S*)-**RuNCS** (bottom) and on (*R*)-**RuNCS** (top) assembled on a magnetic AuNi^{TS} electrode. The (*R*)-**RuNCS** enantiomer displays broader statistical dispersion across independent junction realizations, nevertheless the ensemble mean consistently captures the CISS-induced rectification asymmetry between H_{UP} and H_{DOWN} configurations for both enantiomers.

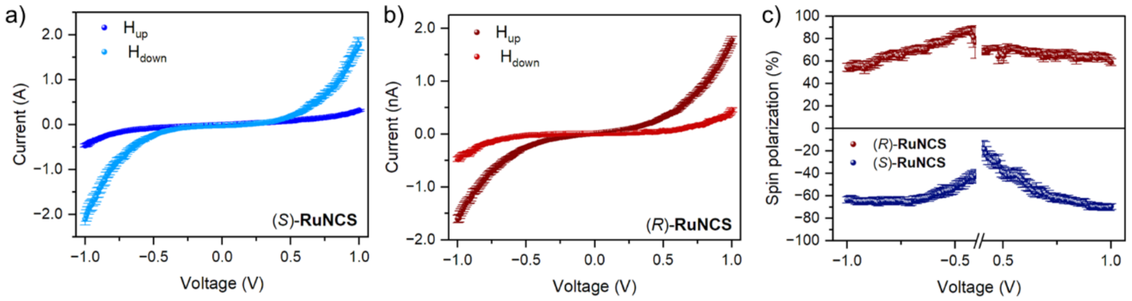

Figure S33. Averaged room temperature *I*/*V* curves acquired on (a) AuNi^{TS}(*S*)-**RuNCS** and on (b) AuNi^{TS}(*R*)-**RuNCS** under constant ±0.2 T magnetic field (H_{UP} and H_{DOWN} orientations); (c) Spin polarization percentage extracted from the corresponding *I*/*V* curve for SAMs of both enantiomers at room temperature under constant ±0.2 T magnetic Field. The error bars correspond to the standard error $\sigma/\sqrt{n}$ where σ is the standard deviation and n is the number of measurements.

References

(1) Dolomanov, O. V.; Bourhis, L. J.; Gildea, R. J.; Howard, J. A. K.; Puschmann, H. *OLEX2*: A Complete Structure Solution, Refinement and Analysis Program. *J. Appl. Crystallogr.* **2009**, *42* (2), 339–341. https://doi.org/10.1107/S0021889808042726.

(2) Sheldrick, G. M. *SHELXT* – Integrated Space-Group and Crystal-Structure Determination. *Acta Crystallogr. Sect. Found. Adv.* **2015**, *71* (1), 3–8. https://doi.org/10.1107/S2053273314026370.

(3) Sheldrick, G. M. Crystal Structure Refinement with *SHELXL*. *Acta Crystallogr. Sect. C Struct. Chem.* **2015**, *71* (1), 3–8. https://doi.org/10.1107/S2053229614024218.

(4) Vogel, N.; Zieleniecki, J.; Köper, I. As Flat as It Gets: Ultrasmooth Surfaces from Template-Stripping Procedures. *Nanoscale* **2012**, *4* (13), 3820. https://doi.org/10.1039/c2nr30434a.

(5) Coloma, I.; Buffeteau, T.; Pecastaings, G.; Herrero, S.; Hillard, E.; Rosa, P.; Cortijo, M.; Gonidec, M. Robust Large-Area Molecular Junctions of Self-Assembled Monolayers of a Model Helical Paddlewheel Complex. *Nanoscale* **2025**, *17* (19), 12065–12071. https://doi.org/10.1039/D5NR00050E.

(6) Buffeteau, T.; Desbat, B.; Turlet, J. M. Polarization Modulation FT-IR Spectroscopy of Surfaces and Ultra-Thin Films: Experimental Procedure and Quantitative Analysis. *Appl. Spectrosc.* **1991**, *45* (3), 380–389. https://doi.org/10.1366/0003702914337308.

(7) Buffeteau, T.; Desbat, B.; Blaudez, D.; Turlet, J. M. Calibration Procedure to Derive IRRAS Spectra from PM-IRRAS Spectra. *Appl. Spectrosc.* **2000**, *54* (11), 1646–1650. https://doi.org/10.1366/0003702001948673.

(8) Ramin, M. A.; Le Bourdon, G.; Daugey, N.; Bennetau, B.; Vellutini, L.; Buffeteau, T. PM-IRRAS Investigation of Self-Assembled Monolayers Grafted onto SiO_2/Au Substrates. *Langmuir* **2011**, *27* (10), 6076–6084. https://doi.org/10.1021/la2006293.

(9) Anthony, M. T.; Seah, M. P. XPS: Energy Calibration of Electron Spectrometers. 1—An Absolute, Traceable Energy Calibration and the Provision of Atomic Reference Line Energies. *Surf. Interface Anal.* **1984**, *6* (3), 95–106. https://doi.org/10.1002/sia.740060302.

(10) Yeh, J. J.; Lindau, I. Atomic Subshell Photoionization Cross Sections and Asymmetry Parameters: $1 \leqslant Z \leqslant 103$. *At. Data Nucl. Data Tables* **1985**, *32* (1), 1–155. https://doi.org/10.1016/0092-640X(85)90016-6.

(11) Frisch, M. J.; Trucks, G. W.; Schlegel, H. B.; Scuseria, G. E.; Robb, M. A.; Cheeseman, J. R.; Scalmani, G.; Barone, V.; Mennucci, B.; Petersson, G. A.; Nakatsuji, H.; Caricato, M.; Li, X.; Hratchian, H. P.; Izmaylov, A. F.; Bloino, J.; Zheng, G.; Sonnenberg, J. L.; Hada, M.; Ehara, M.; Toyota, K.; Fukuda, R.; Hasegawa, J.; Ishida, M.; Nakajima, T.; Honda, Y.; Kitao, O.; Nakai, H.; Vreven, T.; Montgomery, J. A.; Peralta, J. E.; Ogliaro, F.; Bearpark, M.; Heyd, J. J.; Brothers, E.; Kudin, K. N.; Staroverov, V. N.; Kobayashi, R.; Normand, J.; Raghavachari, K.; Rendell, A.; Burant, J. C.; Iyengar, S. S.; Tomasi, J.; Cossi, M.; Rega, N.; Millam, J. M.; Klene, M.; Knox, J. E.; Cross, J. B.; Bakken, V.; Adamo, C.; Jaramillo, J.; Gomperts, R.; Stratmann, R. E.; Yazyev, O.; Austin, A. J.; Cammi, R.; Pomelli, C.; Ochterski, J. W.; Martin, R. L.; Morokuma, K.; Zakrzewski, V. G.; Voth, G. A.; Salvador, P.; Dannenberg, J. J.; Dapprich, S.; Daniels, A. D.; Farkas; Foresman, J. B.; Ortiz, J. V.; Cioslowski, J.; Fox, D. J.; Montgomery J. A., Jr.; Peralta, J. E.; Ogliaro, F.; Bearpark, M.; Heyd, J. J.; Brothers, E.; Kudin, K. N.; Staroverov, V. N.; Kobayashi, R.; Normand, J.; Raghavachari, K.; Rendell, A.; Burant, J. C.; Iyengar, S. S.; Tomasi, J.; Cossi, M.; Rega, N.; Millam, N. J.; Klene, M.; Knox, J. E.; Cross, J. B.; Bakken, V.; Adamo, C.; Jaramillo, J.; Gomperts, R.; Stratmann, R. E.; Yazyev, O.; Austin, A. J.; Cammi, R.; Pomelli, C.; Ochterski, J. W.; Martin, R. L.; Morokuma, K.; Zakrzewski, V. G.; Voth, G. A.; Salvador, P.; Dannenberg, J. J.; Dapprich, S.; Daniels, A.

D.; Farkas, Ö.; Foresman, J. B.; Ortiz, J. V.; Cioslowski, J.; Fox, D. J. *Gaussian 09, Revision A.1*; Gaussian, Inc.: Wallingford, CT, 2009.
(12) Bowers, C. M.; Liao, K.-C.; Yoon, H. J.; Rappoport, D.; Baghbanzadeh, M.; Simeone, F. C.; Whitesides, G. M. Introducing Ionic and/or Hydrogen Bonds into the SAM//Ga_2O_3 Top-Interface of $Ag^{TS}/S(CH_2)_nT//Ga_2O_3$/EGaIn Junctions. *Nano Lett.* **2014**, *14* (6), 3521–3526. https://doi.org/10.1021/nl501126e.
(13) Baghbanzadeh, M.; Bowers, C. M.; Rappoport, D.; Żaba, T.; Gonidec, M.; Al-Sayah, M. H.; Cyganik, P.; Aspuru-Guzik, A.; Whitesides, G. M. Charge Tunneling along Short Oligoglycine Chains. *Angew. Chem. Int. Ed.* **2015**, *54* (49), 14743–14747. https://doi.org/10.1002/anie.201507271.
(14) Simeone, F. C.; Yoon, H. J.; Thuo, M. M.; Barber, J. R.; Smith, B.; Whitesides, G. M. Defining the Value of Injection Current and Effective Electrical Contact Area for EGaIn-Based Molecular Tunneling Junctions. *J. Am. Chem. Soc.* **2013**, *135* (48), 18131–18144. https://doi.org/10.1021/ja408652h.
